# Acetylcholinesterase inhibition protects against trastuzumab-induced cardiotoxicity through reducing multiple programmed cell death pathways

**DOI:** 10.1186/s10020-023-00686-7

**Published:** 2023-09-11

**Authors:** Thawatchai Khuanjing, Chayodom Maneechote, Benjamin Ongnok, Nanthip Prathumsap, Apiwan Arinno, Titikorn Chunchai, Busarin Arunsak, Siriporn C. Chattipakorn, Nipon Chattipakorn

**Affiliations:** 1 Cardiac Electrophysiology Research and Training Center, Faculty of Medicine, Chiang Mai University, Chiang Mai, 50200 Thailand; 2 Cardiac Electrophysiology Unit, Department of Physiology, Faculty of Medicine, Chiang Mai University, Chiang Mai, 50200 Thailand; 3 Center of Excellence in Cardiac Electrophysiology Research, Chiang Mai University, Chiang Mai, 50200 Thailand; 4 Department of Oral Biology and Diagnostic Sciences, Faculty of Dentistry, Chiang Mai University, Chiang Mai, 50200 Thailand

**Keywords:** Acetylcholinesterase inhibitor, Trastuzumab, Cardiotoxicity, Mitochondria, Programmed cell death pathways

## Abstract

**Background:**

Trastuzumab (Trz)-induced cardiotoxicity (TIC) is one of the most common adverse effects of targeted anticancer agents. Although oxidative stress, inflammation, mitochondrial dysfunction, apoptosis, and ferroptosis have been identified as potential mechanisms underlying TIC, the roles of pyroptosis and necroptosis under TIC have never been investigated. It has been shown that inhibition of acetylcholinesterase function by using donepezil exerts protective effects in various heart diseases. However, it remains unknown whether donepezil exerts anti-cardiotoxic effects in rats with TIC. We hypothesized that donepezil reduces mitochondrial dysfunction, inflammation, oxidative stress, and cardiomyocyte death, leading to improved left ventricular (LV) function in rats with TIC.

**Methods:**

Male Wistar rats were randomly assigned to be Control or Trz groups (Trz 4 mg/kg/day, 7 days, I.P.). Rats in Trz groups were assigned to be co-treated with either drinking water (Trz group) or donepezil 5 mg/kg/day (Trz + DPZ group) via oral gavage for 7 days. Cardiac function, heart rate variability (HRV), and biochemical parameters were evaluated.

**Results:**

Trz-treated rats had impaired LV function, HRV, mitochondrial function, and increased inflammation and oxidative stress, leading to apoptosis, ferroptosis, and pyroptosis. Donepezil co-treatment effectively decreased those adverse effects of TIC, resulting in improved LV function. An in vitro study revealed that the cytoprotective effects of donepezil were abolished by a muscarinic acetylcholine receptor (mAChR) antagonist.

**Conclusions:**

Donepezil exerted cardioprotection against TIC via attenuating mitochondrial dysfunction, oxidative stress, inflammation, and cardiomyocyte death, leading to improved LV function through mAChR activation. This suggests that donepezil could be a novel intervention strategy in TIC.

**Supplementary Information:**

The online version contains supplementary material available at 10.1186/s10020-023-00686-7.

## Background

Trastuzumab (Trz) is a recombinant humanized monoclonal antibody, which effectively reduces the mortality in various malignancies including patients with breast cancer, gastric cancer, and colorectal cancer by inhibiting the human epidermal growth factor receptor 2 (HER2) (Alsina et al. 2022; Vega Cano et al. 2022; Bando et al. 2023). Unfortunately, Trz-induced cardiotoxicity (TIC) has emerged as a serious adverse effect of Trz (Banke et al. 2019; Gorini et al. 2018). Oxidative stress, inflammation, and cardiac mitochondrial dysfunction have been identified as potential mechanisms underlying the deleterious consequences of TIC (Varga et al. 2015; Choksey and Timm 2021; Ma et al. 2020). TIC is related to the blockage of the HER2 signaling pathway in cardiomyocytes, resulting in increased oxidative stress and impaired cardiac function (Wu et al. 2022). Additionally, Trz treatment increased inflammatory response and mononuclear cell infiltration through activation of Toll-like receptor 4 (Yousif and Al-amran 2011). Trz also dysregulated cellular metabolism by reducing AMP-activated protein kinase (AMPK) activation, mitochondrial respiration, and cellular adenosine triphosphate in human-induced pluripotent stem cell-derived cardiomyocytes (Kitani et al. 2019). Inhibition of AMPK by Trz upregulated the mammalian target of rapamycin (mTOR) and subsequently suppressed autophagy activity (Kitani et al. 2019).

Cardiac autonomic dysfunction has been associated with TIC (Lenneman et al. 2014; Guimaraes et al. 2015). In HER2^+^ breast cancer patients receiving Trz, norepinephrine and systolic blood pressure were increased, suggesting sympathetic hyperactivity (Lenneman et al. 2014). This sympathetic hyperactivity in TIC was involved with the modulation of the rostral ventrolateral medulla, resulting in an increased sympathetic outflow (Lenneman et al. 2014; Matsukawa et al. 2011). Although modulation of cardiac autonomic activity using acetylcholinesterase (AChE) inhibitors, including donepezil and pyridostigmine, has been demonstrated to exert cardioprotection in many heart diseases (Khuanjing et al. 2020a, 2021a, 2021b; Lu et al. 2018), the anti-cardiotoxic effects of donepezil against TIC remain largely unknown.

Cardiomyocyte death is a significant process during the progression of the TIC (Ma et al. 2020; Sun et al. 2022; Kabel and Elkhoely 2017; Riccio et al. 2018). Apoptosis, a well-known programmed cell death, was markedly increased in both Trz-treated mice and neonatal rat ventricular myocytes as evidenced by increased expression of Bcl-2-associated x protein (Bax), cytochrome c release, and activation of caspases, while decreased anti-apoptotic B-cell lymphoma-extra-large (Bcl-xL) (Kabel and Elkhoely 2017; Riccio et al. 2018). In addition to apoptosis, ferroptosis which is another programmed cell death pathway, which defined by the accumulation of iron and lipid peroxidation, was found to be upregulated following Trz treatment as evidenced by upregulated intracellular iron, reactive oxygen species (ROS) production, Acyl-CoA synthetase long-chain family member 4 (ACSL4), along with the reduced antioxidant enzyme glutathione peroxidase 4 levels in H9c2 cells (Sun et al. 2022; Re et al. 2019). Although both apoptosis and ferroptosis have been identified to be responsible for cell deaths in TIC (Sun et al. 2022; Kabel and Elkhoely 2017; Riccio et al. 2018), other important programmed cell death pathways including necroptosis and pyroptosis in TIC remain unknown.

Necroptosis is a novel programmed cell death pathway, which significantly contributes to the doxorubicin-induced cardiotoxicity (DIC) (Khuanjing et al. 2021a; Yu et al. 2020). Mechanistically, tissue necrotic factor-alpha (TNF-α) binds and activates tissue necrosis factor receptor 1 (TNFR1) (Ying et al. 2021; Mishra et al. 2019), leading to the formation of receptor-interacting protein kinase 1/3 (RIP1/3) which then phosphorylates mixed-lineage kinase domain-like (MLKL) to initiate necroptosis cell death (Ying et al. 2021; Mishra et al. 2019). In addition to necroptosis, pyroptosis is another important programmed cell death, associated with membrane pore formation and extracellular release of pro-inflammatory cytokines that eventually cause inflammation (Re et al. 2019). Pyroptosis is mediated by inflammasome sensors such as the NLR family pyrin domain containing 3 (NLRP3) inflammasome, which can be activated by infection, toxins, or intracellular stresses such as mitochondrial ROS and oxidized mitochondrial DNA (Re et al. 2019; Zhou et al. 2011). NLRP3 inflammasome activates caspase 1, subsequently cleaves Gasdermin D into active forms, leading to Gasdermin D pore formation and membrane rupture (Re et al. 2019). The cell membrane rupture allows the pro-inflammatory cytokines released including interleukin-18 (IL-18) and IL-1β (Re et al. 2019). Currently, the comprehensive roles of these programmed cell death pathways in TIC still need to be clarified.

We determined the roles of donepezil on the improvement of cardiac function and its associated responsible mechanisms including inflammation, oxidative stress, cardiac mitochondrial function, mitochondrial dynamics, and the programmed cell death pathways in rats with TIC. We hypothesized that necroptosis and pyroptosis are involved in the TIC, leading to decreased left ventricular (LV) function. Furthermore, donepezil decreases ROS levels, inflammation, mitochondrial dynamic imbalance, mitochondrial dysfunction, and cardiomyocyte death, resulting in alleviated LV dysfunction in Trz-treated rats.

## Methods

### Animal preparation

Male Wistar rats were purchased from Nomura Siam International Co. Ltd, Bangkok, Thailand. The animals were kept in ventilated cages and maintained on a 12-h light/12-h dark cycle. The G*power program (version 3.1.9.4) was used to calculate the sample size. In addition, our study design was planned based on our preliminary study and previous studies (Riccio et al. 2018; Olorundare et al. 2020; Coppola et al. 2016). The preliminary study showed that 7 days of trastuzumab reduced the percentage of LV ejection fraction and LV fractional shortening with an acceptable mortality rate.

### Experimental design

**Fig. 1:**
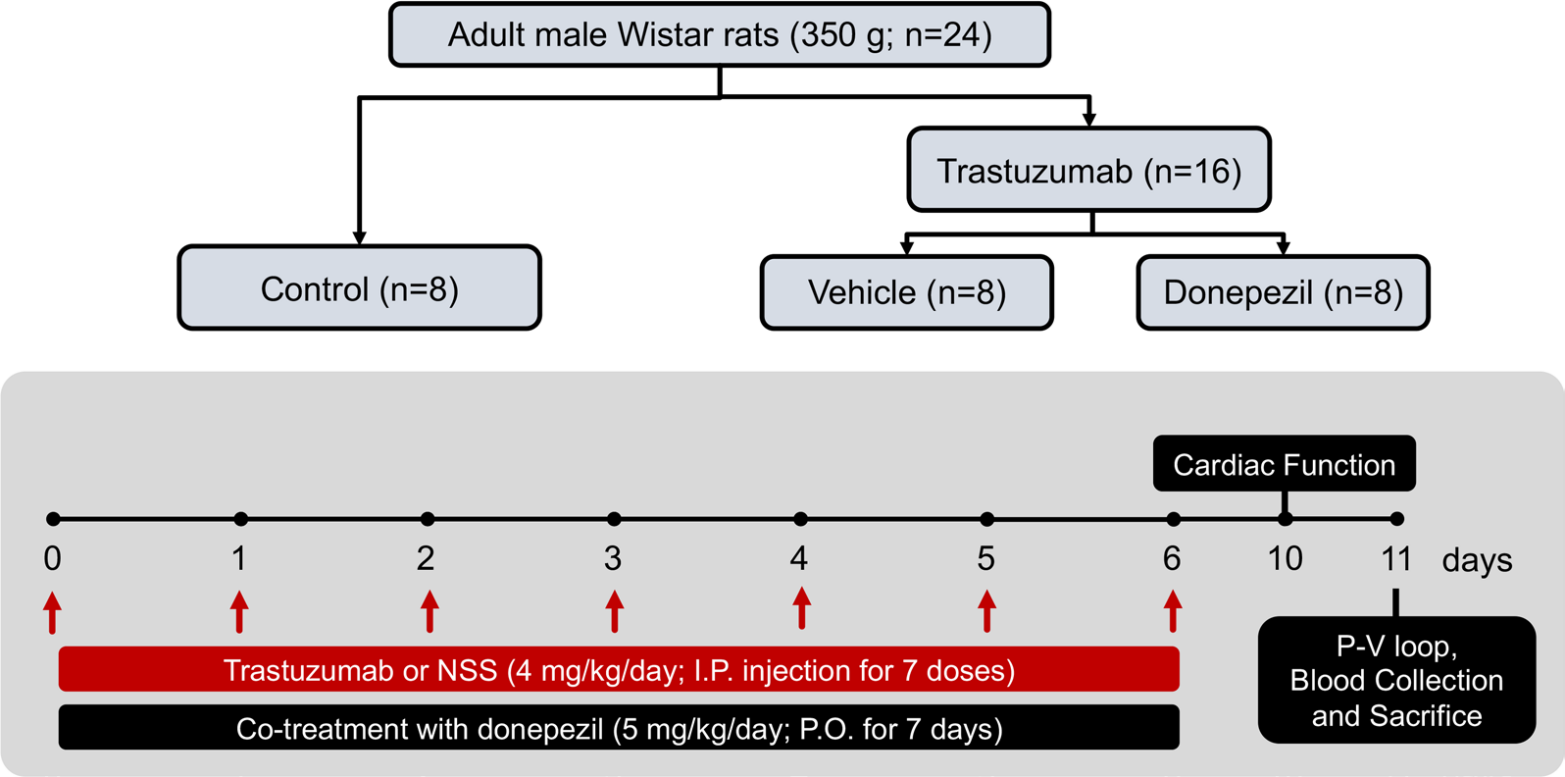
An illustrated diagram of the study protocol. *ip* intraperitoneal injection; *NSS* normal saline solution; *po* oral administration

All rats were acclimatized for 1 week. Rats weighing around 300–350 g were divided into three groups (n = 8/group), including (i) the Control group, (ii) the Trz group, and (iii) the Trz + DPZ group. The Trz-treated rats were intraperitoneally injected with Trz at a dose of 4 mg/kg/day for 7 days. Whereas rats in the Control group received intraperitoneal injections of normal saline solution (NSS). Rats in Trz + DPZ group were treated with donepezil at a dose of 5 mg/kg/day for 7 days via oral gavage, while rats in the Trz group received drinking water. The dose of Trz employed in this study was selected according to the clinical relevance and our pilot study. The dose of donepezil was selected as previously described (Handa et al. 2009). Cardiac function and heart rate variability (HRV) were investigated 4 days after the last Trz injection. Next, invasive LV function was evaluated, followed by blood collection. The rats were euthanized with an overdose of inhaled isoflurane and decapitation. After that, heart tissue was collected to evaluate the cardiac mitochondrial function, mitochondrial morphology, mitochondrial dynamics, apoptosis, pyroptosis, necroptosis, ferroptosis, inflammation, autophagy, mitophagy, and oxidative stress. Furthermore, an ELISA assay was used to determine serum cardiac injury biomarkers. In this study, no animals died during the actual experiments in any of the groups. An illustrated diagram of the experiment is summarized in Fig. 1.

### Echocardiography

LV function was determined under light anesthesia (inhalation of isoflurane, 5% induction, 3% maintenance) using transthoracic echocardiography (Philips, Amsterdam, Netherlands) (Maneechote et al. 2019). %LV ejection fraction (%LVEF), the primary endpoint of this study, was analyzed from M-mode parasternal short-axis view as indicative of systolic function. Furthermore, diastolic function was evaluated by determining the transmitral early filling to atrial filling velocities (E/A) ratio (Maneechote et al. 2019).

### Invasive LV function assessment

In order to evaluate the invasive LV function, a pressure–volume loop system (Transonic Scisense Inc., Ontario, Canada) was utilized as mentioned previously (Maneechote et al. 2019). To anesthetize the rats, an intramuscular injection with a combination of Zoletil (50 mg/kg, Virbac, Carros, France) and Xylazine (0.15 mg/kg, Thai Meiji Pharmaceutical Co., Ltd., Bangkok, Thailand) was performed. The catheter was introduced via the right carotid artery and inserted into the LV. Parameters of the LV function were recorded with Labscribe software (New Hampshire, USA) (Maneechote et al. 2019).

### Cardiac sympathovagal balance assessment

An electrocardiogram (lead II) was obtained using PowerLab 4/25T (ADInstruments, Inc., Australia). RR intervals from a section of the tachogram that contained at least 300 consecutive RR intervals were chosen for HRV analysis according to a previous study (Maneechote et al. 2019). The parasympathetic activity was represented by the high-frequency component (HF), whereas the sympathetic and parasympathetic activities were indicated by the low-frequency component (LF). Sympathovagal balance was determined by calculating the ratio of LF to HF. Sympathetic hyperactivity and/or parasympathetic withdrawal were represented by a higher ratio of LF/HF (Apaijai et al. 2013).

### Cardiac mitochondrial isolation

The heart was promptly dissected, and the heart tissues were washed with cold NSS. Subsequently, the heart tissue was minced and homogenized with an ice-cold isolation buffer. Differential centrifugation was used to isolate mitochondria as previously described (Maneechote et al. 2019). A bicinchoninic acid assay (Sigma-Aldrich, USA) was performed to quantify the concentration of cardiac mitochondria. Isolated cardiac mitochondria were kept cold until analysis.

### Cardiac mitochondrial ROS production

2′,7′-Dichlorohydro-fluorescein diacetate (DCFH-DA) dye (Sigma-Aldrich, USA) was utilized to quantify the cardiac mitochondrial ROS production as mentioned previously (Maneechote et al. 2019). DCF fluorescence signal was evaluated with a microplate reader (BioTek, USA). The fluorescence intensity was directly correlated with the mitochondrial ROS levels.

### Cardiac mitochondrial membrane potential (MMP) measurement

MMP was measured using 5,5′,6,6′-tetrachloro-1,1′,3,3′-tetraethyl-benzimidazolylcarbocyanine iodide (JC-1) dye (Sigma-Aldrich, USA) (Maneechote et al. 2019). In functional mitochondria, the dye forms clusters that fluoresce red. In contrast, the dye exists as a monomer and fluoresces green in defective mitochondria with lower levels of negative MMP. The decreased red to green ratio represents mitochondrial depolarization.

### Cardiac mitochondrial morphology and mitochondrial swelling

Transmission electron microscopy (JEM-1200 EX II, JEOL Ltd., Japan) was utilized to obtain cardiac mitochondrial morphology as described previously (Maneechote et al. 2019). Furthermore, the mitochondrial suspension absorbance was determined to evaluate the mitochondrial swelling using a spectrophotometer (BioTek, USA) (Maneechote et al. 2019). A lower suspension absorbance is indicative of increased mitochondrial swelling.

### Western blot analysis

The heart was promptly dissected and kept at – 80 °C until sample preparation. Sample preparation and Western blot analysis were carried out according to a previous study (Maneechote et al. 2019). In brief, after mixing the total protein with the sample loading buffer, the mixture was incubated at 95 °C for 10 min. The protein was separated in 10% or 12.5% SDS–polyacrylamide gel. After that, the protein was transferred to a nitrocellulose membrane. 5% skim milk or 5% bovine serum albumin was used for blocking the membrane at RT for 1 h. Subsequently, the primary antibody was applied and kept at 4 °C overnight. The membrane was then probed with a secondary antibody and visualized with chemiluminescence imaging systems (Bio-Rad Laboratories, CA, USA). Additional file 1: Table S1 lists all antibodies utilized in this study. ImageJ software (version 1.53a) was used to quantify protein expression.

### Quantification of cardiac injury biomarkers

ELISA assay kits (MyBioSource, USA) were utilized to quantify the levels of cardiac troponin I (cTnI) and N-terminal pro b-type natriuretic peptide (NT-proBNP) in the serum. The absorbance at 450 nm was evaluated using a spectrophotometer (BioTek, USA).

### Malondialdehyde (MDA) measurement

Cardiac and serum MDA levels were quantified using the HPLC system (Thermo Fisher Scientific, USA), in accordance with a previous study (Maneechote et al. 2019). The cardiac tissue and serum samples were incubated with 10% trichloroacetic acid at 90 °C for 30 min. Following centrifugation, the supernatant was transferred to a solution of thiobarbituric acid and phosphoric acid, then incubated at 90 °C for 30 min. Thiobarbituric acid-reactive substances (TBARS) were produced as a product of MDA. The TBARS levels were measured at 532 nm.

### RT-qPCR

Cardiac tissue was quickly removed from rats and kept at − 80 °C in RNAlater™ stabilizing solution (Invitrogen, USA) until analysis. To isolate total RNAs, the TRIzolTM Reagent (Thermo Fisher Scientific, Waltham, MA, USA) was used. Then, single-stranded complementary DNA (cDNA) was produced using the iScript cDNA Synthesis Kit (Bio-Rad, California, USA). After that, cDNA was diluted with RNase-free water. The SsoFast™ EvaGreen Supermix Kit and the Bio-Rad Cx96 Detection System (Bio-Rad, USA) were used for the RT-qPCR assay. The details of the primers are indicated in Additional file 1: Table S2. Relative target gene expression was determined using the 2^−ΔΔct^ method.

### TUNEL assay

A TUNEL assay kit (Roche, Switzerland) was utilized to determine cardiomyocyte death, according to the manufacturer’s guidelines (Khuanjing et al. 2021a). TUNEL-positive nuclei, which were stained in red were visualized using a confocal microscope (Olympus, Japan). Additionally, the cells were counterstained with 4′,6-diamidino-2-phenylindole (DAPI), as shown in blue.

### Cell culture

Rat cardiomyoblast cells (H9c2 cells) and breast cancer cell lines (MCF-7 and MDA-MB-231) were obtained from the American Type Culture Collection and were maintained according to a previous study (Khuanjing et al. 2021a).

### Cell viability

3-(4,5-Dimethylthiazol-2-yl)-2,5-diphenyltetrazolium bromide (MTT, Invitrogen, USA), was utilized to measure the vitality of cardiomyocytes and cancer cell lines as described previously (Khuanjing et al. 2021a). The cells (P5-P10) were plated in a 96-well plate (5000 cells/well) and kept in an incubator at 37 °C for 24 h. Trz (8 μM) and varying concentrations of donepezil were applied to the cells and cultured for 48 h in an incubator at 37 °C. Then, the MTT solution was added and kept in an incubator at 37 °C for 4 h. Subsequently, the supernatant was then discarded, and dimethyl sulfoxide was applied to dissolve MTT formazan (RCI Labscan, Thailand). A spectrophotometer (BioTek, USA) was utilized to determine the absorbance. Additionally, atropine, a muscarinic acetylcholine receptor (mAChR) blocker, was used at a concentration of 0.1 mM to assess the role of mAChR on the cytoprotective effects of donepezil in Trz-treated H9c2 cells.

### Cytotoxicity assay

To determine cellular cytotoxicity, lactate dehydrogenase (LDH) release was quantified using the Pierce™ LDH Cytotoxicity Assay Kit (Thermo Fisher Scientific, USA). The cells (5000 cells/well) were plated in a 96-well plate. After 24 h incubation, Trz at a concentration of 8 μM and donepezil at a concentration of 0.5 and 1.0 μM were added and cultured in an incubator for 48 h. Subsequently, the supernatant was mixed with the reaction mixture in a new 96-well plate. After 30 min of incubation, the stop solution was applied, and the absorbance was quantified using a spectrophotometer (BioTek, USA). The absorbance is directly proportional to the cytotoxicity (Russo et al. 2019).

### Determination of AChE activity

The Acetylcholinesterase Assay Kit (Abcam, UK) was used to determine serum AChE activity according to the manufacturer’s guidelines. Briefly, the acetylthiocholine reaction mix was incubated with the serum in the 96-well plate. A spectrophotometer (BioTek, USA) was utilized to measure the absorbance at 410 nm.

### Statistical analysis

All data are presented as the mean ± standard error of the mean. In order to evaluate statistical analysis, GraphPad Prism 8.2.1 software was utilized. To analyze the differences between groups, a one-way analysis of variance (ANOVA) was utilized, followed by an LSD post hoc test. A *p-value* less than 0.05 was considered statistically significant.

## Results

### Donepezil improved LV function and cardiac sympathovagal balance in rats with TIC

**Fig. 2:**
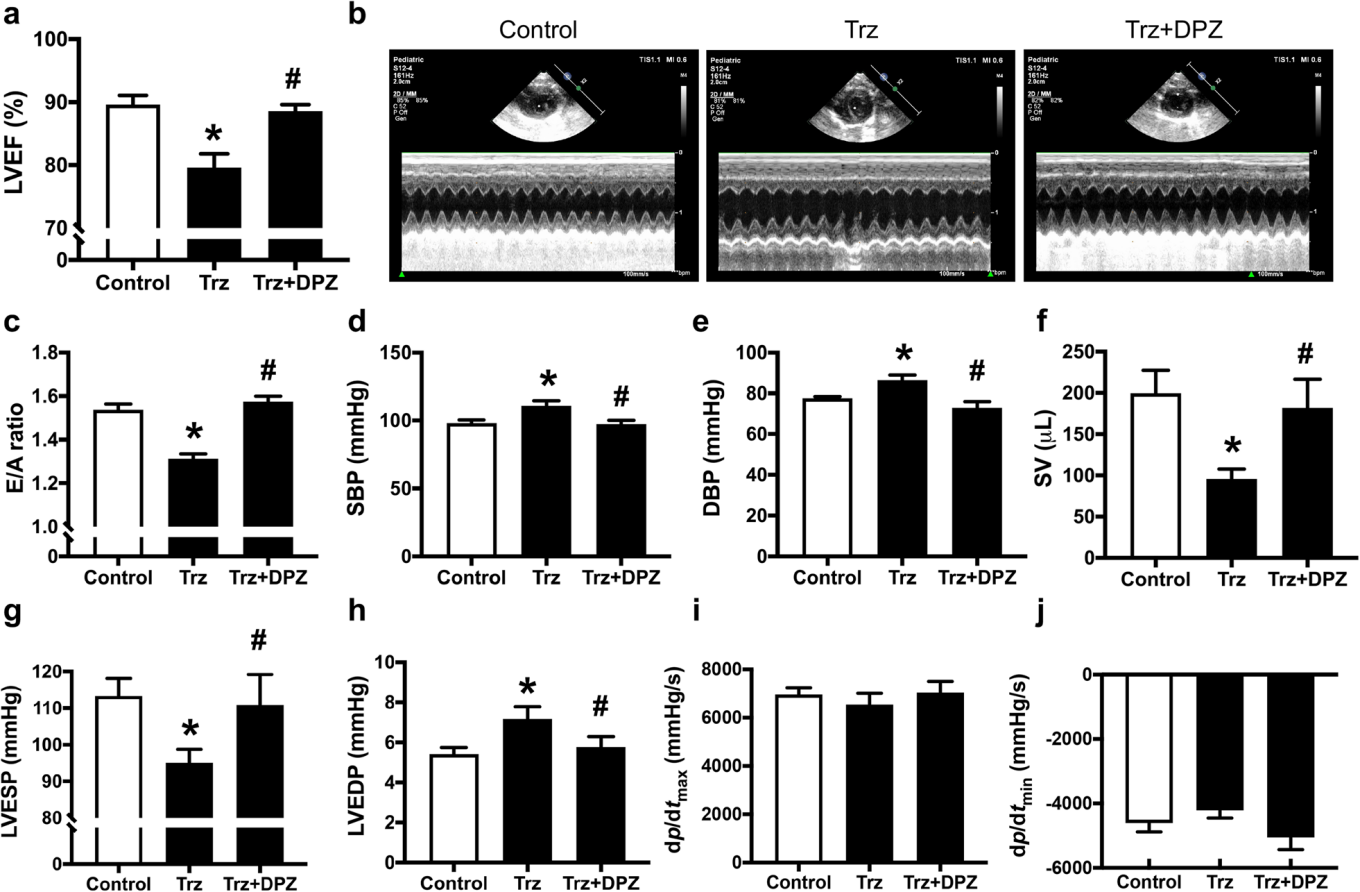
The effects of donepezil on left ventricular function and blood pressure in TIC rats. **a** LVEF; **b** Representative images of echocardiography; **c** E/A ratio; **d** SBP; **e** DBP; **f** SV; **g** LVESP; **h** LVEDP; **i** d*p*/d*t*_max_; **j** d*p*/d*t*_min_. *DBP* diastolic blood pressure; *DPZ* donepezil; *dp/dt*_*max*_ the maximum time derivative of LV pressure; *dp/dt*_*min*_ the minimum time derivative of LV pressure; *E/A* early filling to atrial filling velocities; *SBP* systolic blood pressure; *SV* stroke volume; *Trz* trastuzumab. *n* = 6–8 rats per group. Data are presented as the mean ± SEM; *p < 0.05 vs. control; ^#^p < 0.05 vs. Trz

Trz impaired LV systolic and diastolic function as evidenced by reduced %LVEF and E/A ratio, respectively, compared to the control rats (Fig. 2a–c). Trz also markedly increased systolic and diastolic blood pressure when compared with the control rats (Fig. 2d, e). A pressure–volume loop analysis demonstrated that Trz treatment markedly decreased stroke volume and LV end-systolic pressure, while increasing LV end-diastolic pressure in comparison with the control group (Fig. 2f–h). No alteration in d*p*/d*t*_max_ and d*p*/d*t*_min_ was observed in all groups (Fig. 2i, j). Importantly, donepezil treatment effectively attenuated LV dysfunction in those TIC rats (Fig. 2a–h).

**Fig. 3:**
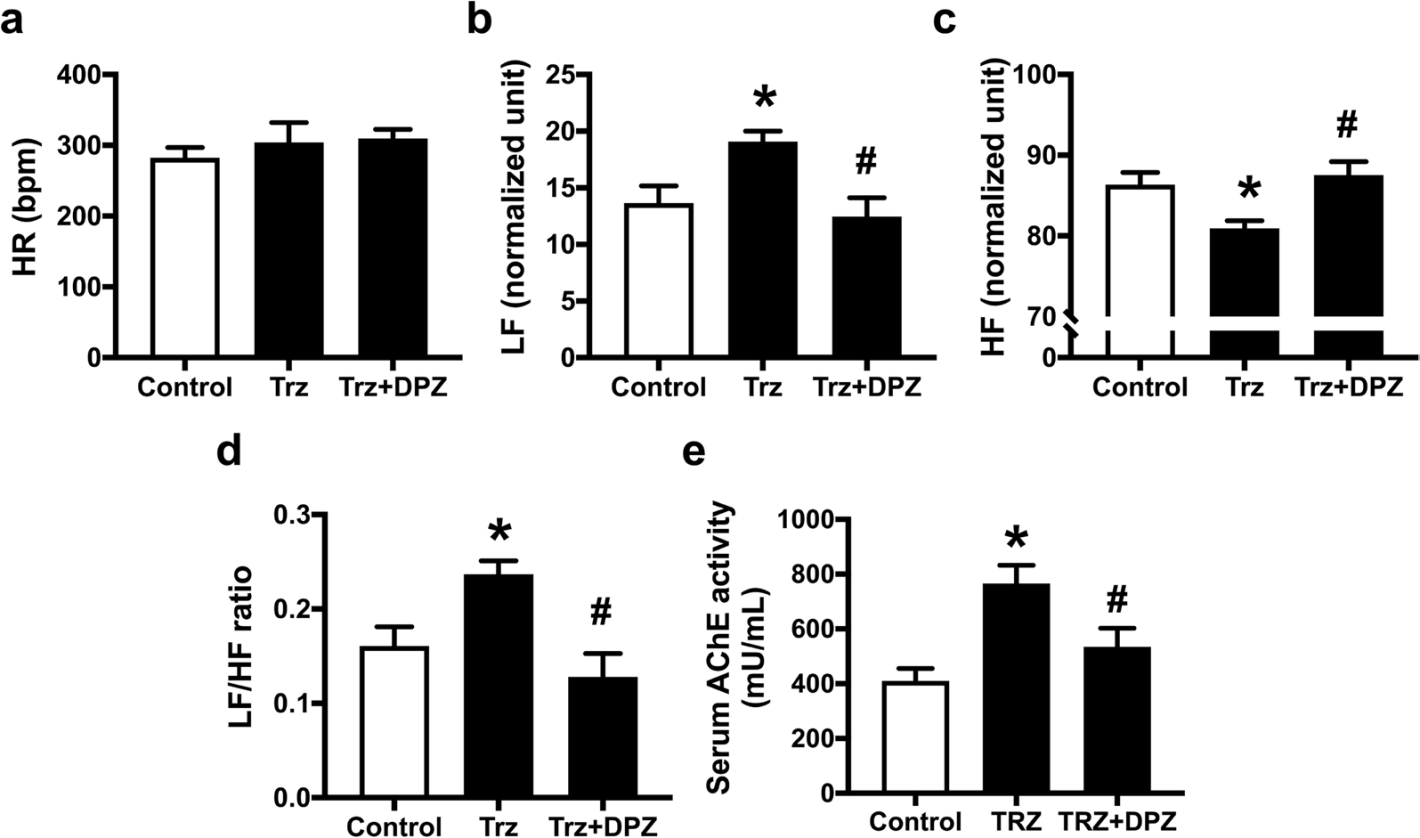
The effects of donepezil on cardiac autonomic function in rats treated with Trz. **a** HR; **b** LF (normalized unit); **c** HF (normalized unit); **d** LF/HF ratio; **e** Serum AChE activity. *AChE* acetylcholinesterase; *DPZ* donepezil; *HF* high-frequency component; *HR* heart rate; *LF* low-frequency component; *Trz* trastuzumab. *n* = 6–8 rats per group. Data are presented as the mean ± SEM; *p < 0.05 vs. control; ^#^p < 0.05 vs. Trz

Although heart rate was not changed in Trz-treated rats (Fig. 3a), the results demonstrated that Trz impaired cardiac autonomic function as evidenced by increased LF (normalized unit) and LF/HF ratio (Fig. 3b, d). Additionally, the HF (normalized unit) was markedly reduced in the Trz-treated rats (Fig. 3c). These results indicated that Trz impaired cardiac sympathovagal balance in TIC rats. We also found that Trz administration markedly increased the activity of AChE activity (Fig. 3e). Interestingly, donepezil activated parasympathetic activity by inhibiting AChE activity, resulting in improved cardiac autonomic function in TIC rats (Fig. 3b–e). These results indicated that donepezil improved cardiac sympathovagal balance by inhibiting AChE in rats with TIC.

### Donepezil decreased cardiac injury, oxidative stress, and inflammation in Trz-treated rats

**Fig. 4:**
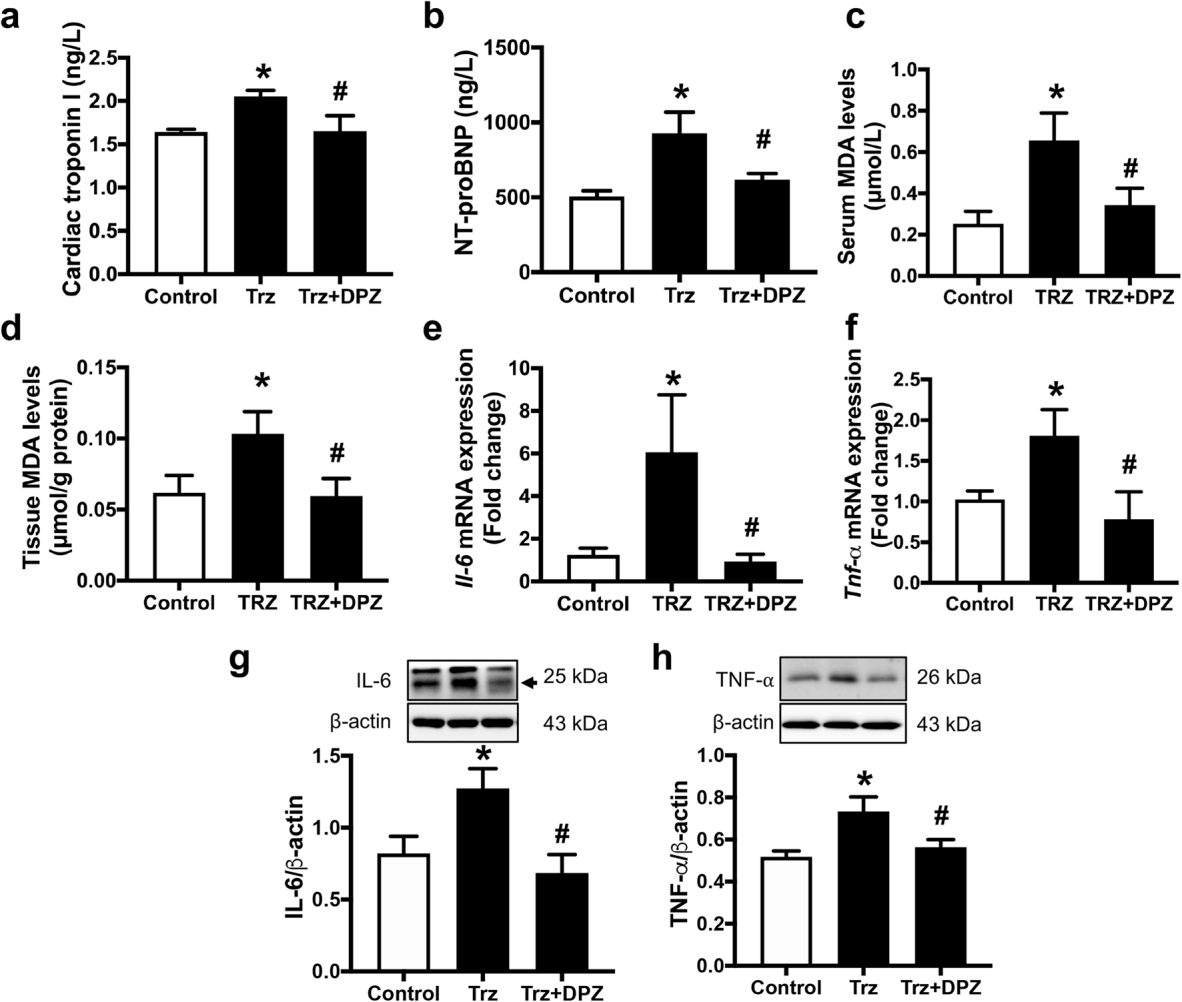
The effects of donepezil on cardiac injury, oxidative stress, and inflammation in TIC rats. **a** Cardiac troponin I levels; **b** NT-proBNP levels; **c** Serum MDA levels; **d** Tissue MDA levels; **e** *Il-6* mRNA expression; **f** *Tnf-α* mRNA expression; **g** IL-6 protein expression; **h** TNF-⍺ protein expression. *DPZ* donepezil; *Il-6* interleukin-6; *MDA* malondialdehyde; *NT-proBNP* N-terminal pro B-type natriuretic peptide; *Tnf-α* tumor necrosis factor-alpha; *Trz* trastuzumab. *n* = 6–8 rats per group. Data are presented as the mean ± SEM; *p < 0.05 vs. control; ^#^p < 0.05 vs. Trz

We then investigated whether donepezil affected cardiomyocyte injury, oxidative stress, and inflammation upon Trz treatment (Fig. 4a–h). Our results showed that both cTnI and NT-proBNP were markedly increased in TIC rats when compared with the control group (Fig. 4a, b). Furthermore, Trz-treated rats significantly increased the MDA levels in both serum and heart tissue compared with the control group (Fig. 4c, d). In addition, Trz rats had significantly higher TNF-α and IL-6 mRNA levels than the control group (Fig. 4e, f). The Western blot results revealed that the expression of IL-6 and TNF-⍺ was significantly increased after Trz treatment, compared to the control rats (Fig. 4g, h). However, co-treatment with donepezil effectively alleviated the expression of these cytokines in Trz-treated rats (Fig. 4e–h). These findings suggested that Trz increased oxidative stress and inflammation, resulting in cardiomyocyte injury. Fortunately, donepezil treatment reduced cardiac oxidative stress and inflammation, leading to reduced cardiomyocyte injury (Fig. 4a–h). These results demonstrated that donepezil effectively reduced cardiomyocyte injury by lowering both oxidative stress and cardiac inflammation.

### Donepezil improved cardiac mitochondrial function and mitochondrial dynamic balance in Trz-treated rats

**Fig. 5:**
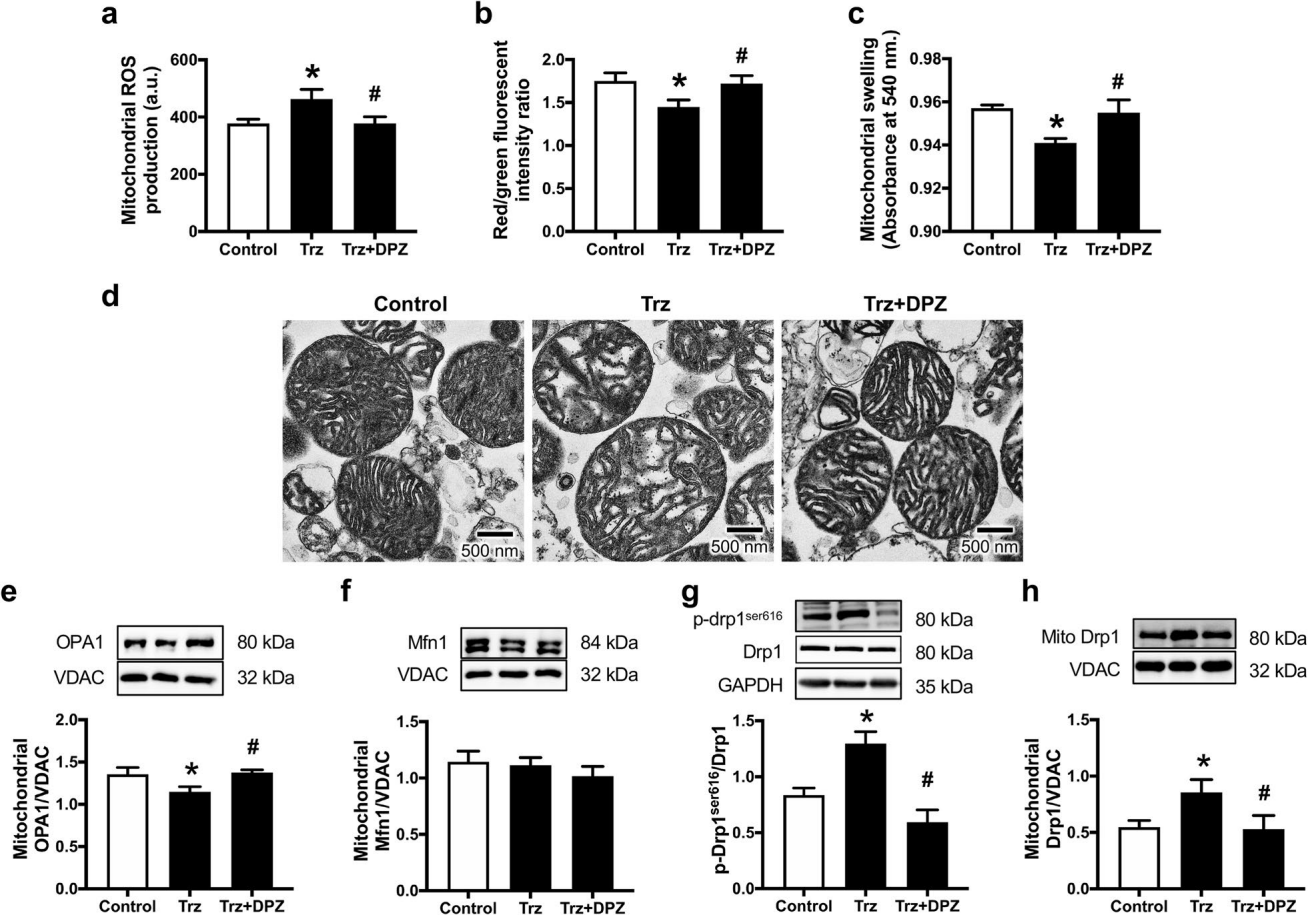
The effects of donepezil on cardiac mitochondrial function and dynamics in TIC rats. **a** Mitochondrial ROS production; **b** Mitochondrial depolarization; **c** Mitochondrial swelling; **d** Mitochondrial morphology; **e** Mitochondrial OPA1 expression; **f** Mitochondrial Mfn1 expression; **g** phosphorylation of Drp1 at serine 616 to total Drp1 ratio; **h** Mitochondrial Drp1 expression. *a.u.* arbitrary units; *Drp1* dynamin-related protein 1; *DPZ* donepezil; *Mfn1* mitofusin 1; *OPA1* optic atrophy 1; *Trz* trastuzumab; *ROS* reactive oxygen species; *VDAC* voltage-dependent anion channel. *n* = 6–8 rats per group. Data are presented as the mean ± SEM; *p < 0.05 vs. control; ^#^p < 0.05 vs. Trz

Trz-treated rats developed cardiac mitochondrial dysfunction, as evidenced by higher mitochondrial ROS levels, loss of MMP, and mitochondrial swelling (Fig. 5a–c). Additionally, mitochondrial dysmorphology was found in Trz-treated hearts, as evidenced by the disorganization of cristae and mitochondrial swelling (Fig. 5d). These impairments were attenuated by donepezil treatment (Fig. 5a–d). In addition, Trz impaired cardiac mitochondrial dynamic balance (Fig. 5e–h). Specifically, Trz significantly reduced mitochondrial fusion-related protein mitochondrial optic atrophy 1 (OPA1) (Fig. 5e and Additional file 1: Fig. S1a, b), without changing mitofusin 1 (Mfn1) expression (Fig. 5f and Additional file 1: Fig. S1c, d). In addition, mitochondrial fission was upregulated in Trz-treated rats as indicated by increased phosphorylation of dynamin-related protein 1 (Drp1) at serine 616 and mitochondrial Drp1 expression (Fig. 5g, h and Additional file 1: Fig. S1e–i). Importantly, mitochondrial dynamic imbalance induced by Trz was alleviated by donepezil co-treatment (Fig. 5e–h and Additional file 1: Fig. S1a–i). Collectively, donepezil attenuated the impairments of both cardiac mitochondrial function and mitochondrial dynamics in Trz-treated rats.

### Donepezil attenuated impaired autophagy in rats with TIC

**Fig. 6:**
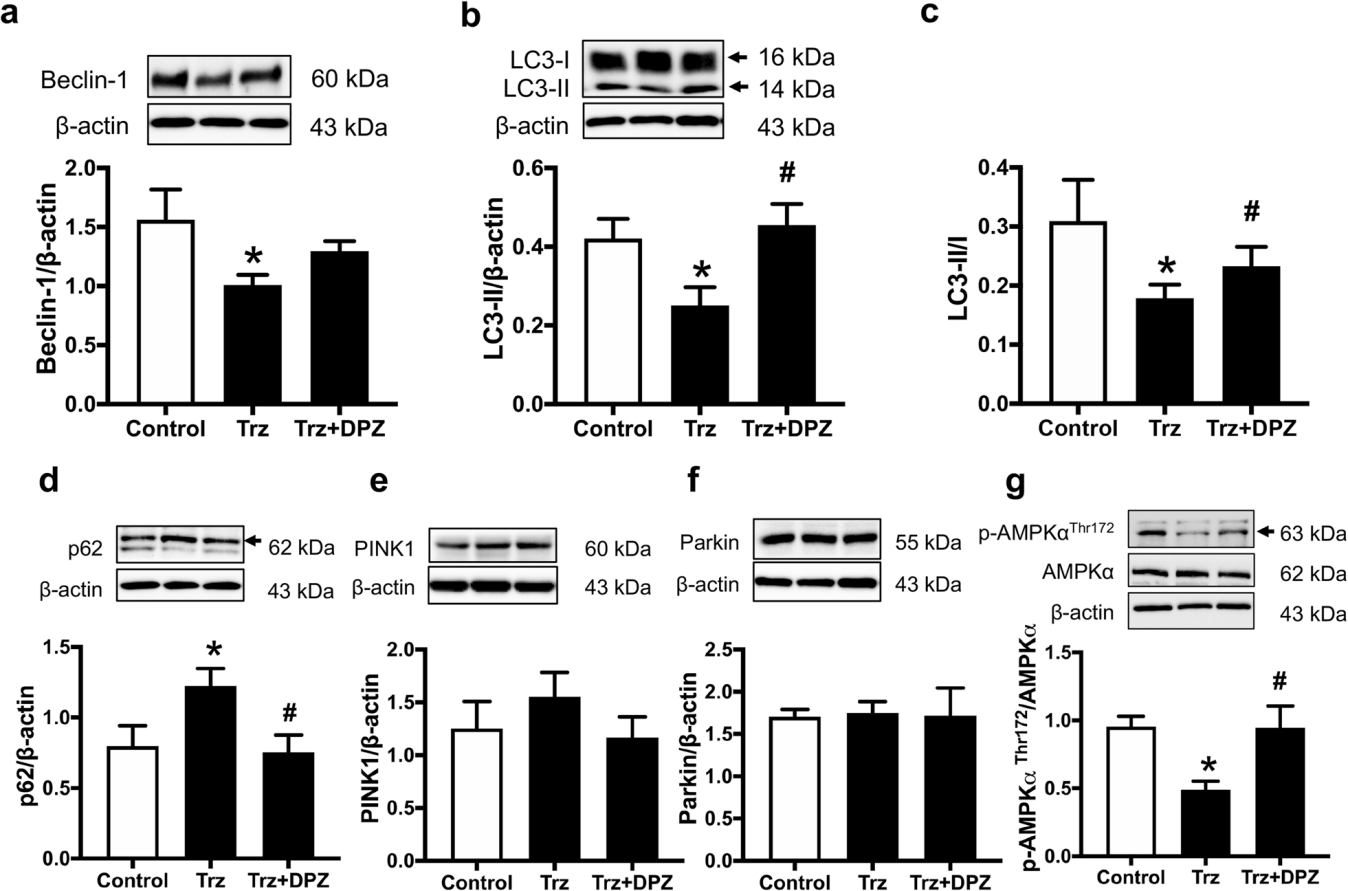
The effects of donepezil on autophagy and mitophagy in TIC rats. **a** Beclin-1 expression; **b** LC3-II expression; **c** LC3-II/I expression; **d** p62 expression; **e** PINK1 expression; **f** Parkin expression; **g** phosphorylation of AMPKα at threonine 172 to total AMPKα ratio. *AMPKα* AMP-activated protein kinase alpha; *DPZ* donepezil; *LC3B-II* light chain 3B-II; *PINK1* PTEN-induced putative kinase 1; *Trz* trastuzumab. *n* = 6–8 rats per group. Data are presented as the mean ± SEM; *p < 0.05 vs. control; ^#^p < 0.05 vs. Trz

Next, we evaluated the role of donepezil on autophagy in Trz-treated hearts. Our results demonstrated that Trz suppressed autophagy initiation and phagophore formation, as evidenced by reduced Beclin-1 expression, LC3-II expression, and LC3-II/I ratio (Fig. 6a–c and Additional file 1: Fig. S2a–d), whereas the sequestosome 1 (p62/SQSTM1) expression was markedly increased in Trz-treated hearts (Fig. 6d and Additional file 1: Fig. S2e, f). These findings suggested that Trz suppressed autophagic flux in rats with TIC. Although donepezil treatment did not change Beclin-1 expression, it markedly increased the expression of LC3-II expression and LC3-II/I ratio, compared with Trz-treated rats (Fig. 6a–c and Additional file 1: Fig. S2a–d). Furthermore, donepezil reduced p62/SQSTM1 expression, suggesting the improvement of autophagy in rats with TIC (Fig. 6d and Additional file 1: Fig. S2e, f). In terms of mitophagy-related proteins, there were no statistical differences among groups (Fig. 6e, f and Additional file 1: Fig. S2g–j). To evaluate the underlying mechanism of donepezil on autophagy, we determined the AMPK expression, an essential upstream signaling pathway of autophagy. Trz-treated hearts showed significantly reduced phosphorylation of AMPK at threonine 172, suggesting the downregulation of AMPK (Fig. 6g and Additional file 1: Fig. S2k–n). However, donepezil treatment restored the phosphorylated AMPK in Trz-treated hearts (Fig. 6g and Additional file 1: Fig. S2k–n).

### Donepezil mitigated cardiomyocyte death by reducing cardiomyocyte apoptosis, pyroptosis, and ferroptosis in rats with TIC

**Fig. 7:**
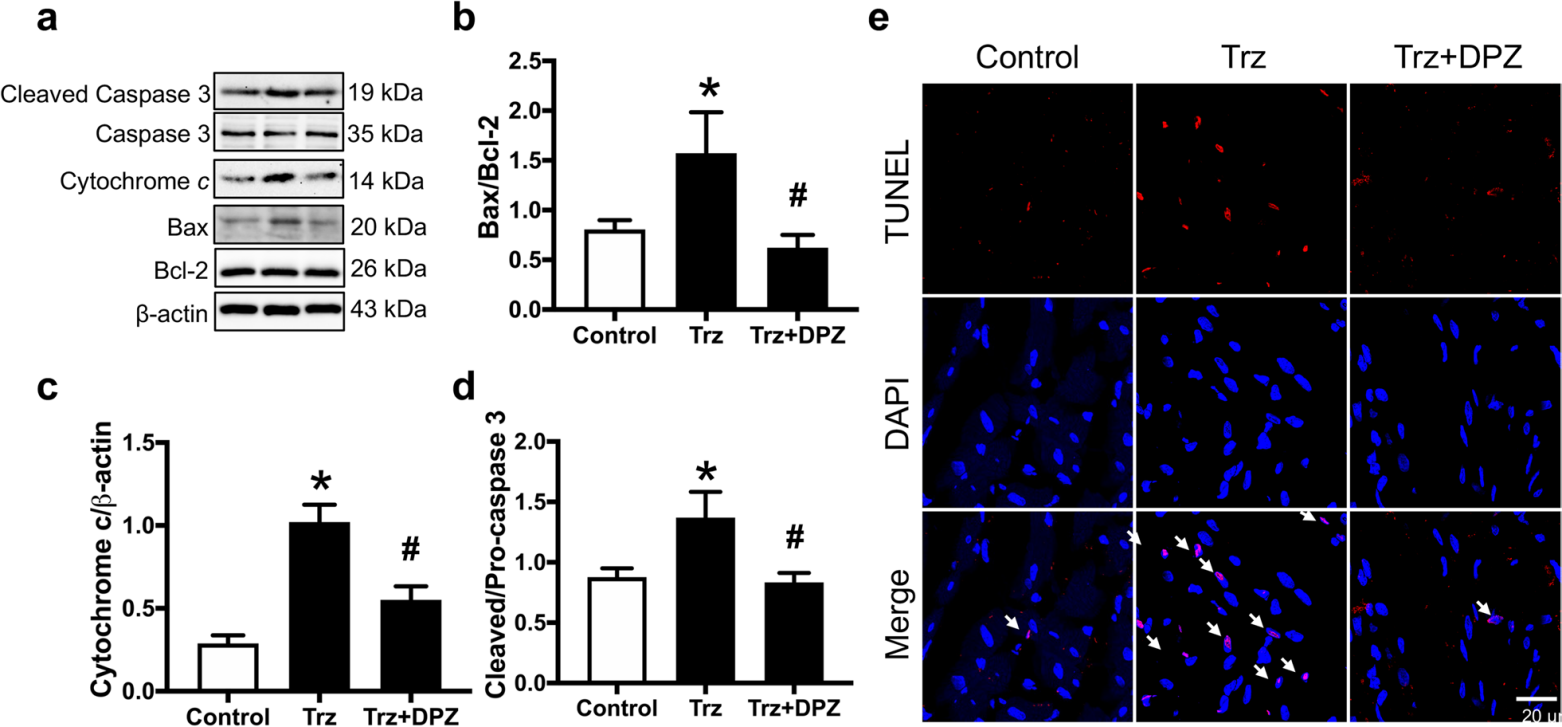
The effects of donepezil on cardiac apoptosis in rats treated with Trz. **a** Representative Western blot bands; **b** Bax/Bcl-2 ratio; **c** Cytochrome *c* expression; **d** Cleaved caspase 3 to pro-caspase 3 ratio; **e** Representative images of TUNEL-positive cells; white arrow indicated the TUNEL-positive cells. *Bax* Bcl-2-associated X; *Bcl-2* B-cell lymphoma 2; *DPZ* donepezil; *Trz* trastuzumab. *n* = 6–8 rats per group. Data are presented as the mean ± SEM; *p < 0.05 vs. control; ^#^p < 0.05 vs. Trz

**Fig. 8:**
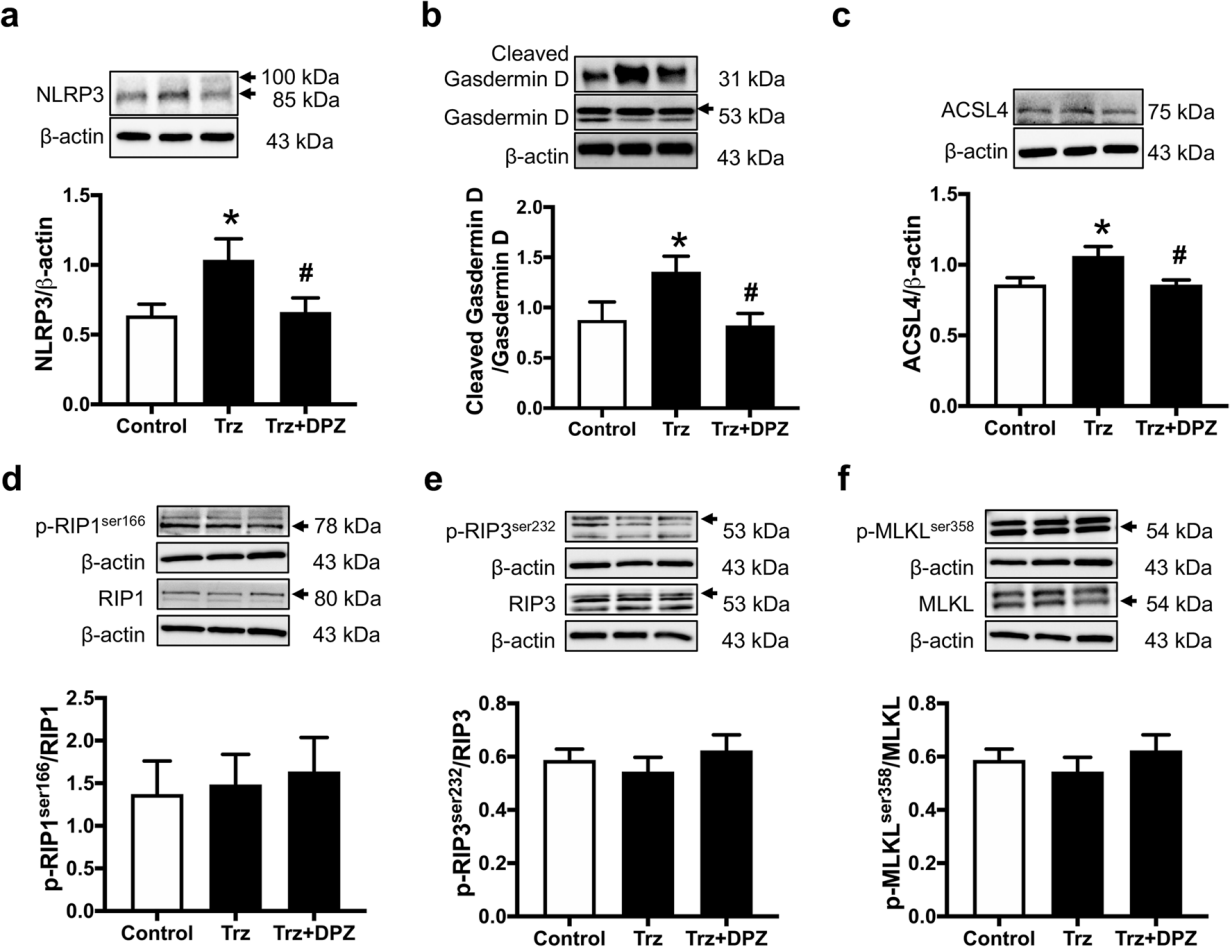
The effects of donepezil on pyroptosis, ferroptosis, and necroptosis in TIC rats. **a** NLRP3 expression; **b** Cleaved Gasdermin D to total Gasdermin D ratio; **c** ACSL4 expression; **d** phosphorylation of RIP1 at serine 166 to RIP1 ratio; **e** phosphorylation of RIP3 at serine 232 to RIP3 ratio; **f** phosphorylation of MLKL at serine 358 to MLKL ratio. *ACSL4* acyl-CoA synthetase long-chain family member 4; *DPZ* donepezil; *MLKL* mixed-lineage kinase domain-like; *NLRP3* NLR family pyrin domain containing 3 inflammasomes; *RIP1* receptor-interacting protein kinase 1; *RIP3* receptor-interacting protein kinase 3; *Trz* trastuzumab. *n* = 6–8 rats per group. Data are presented as the mean ± SEM; *p < 0.05 vs. control; ^#^p < 0.05 vs. Trz

To illustrate the cardioprotective effects of donepezil on cardiomyocyte death in Trz-treated hearts, we determined potential programmed cell death pathways, including apoptosis, pyroptosis, ferroptosis, and necroptosis (Figs. 7, 8 and Additional file 1: Fig. S3, S4). The findings revealed an increase in apoptotic markers in Trz-treated rats, including the ratio of Bax/B-cell lymphoma 2 (Bcl-2), cytochrome c, and the ratio of cleaved caspase 3/caspase 3 (Fig. 7a–d and Additional file 1: Fig. S3a–h). A TUNEL assay was also performed to confirm the presence of apoptotic proteins, as demonstrated in Fig. 7e. Donepezil effectively reduced cardiac apoptosis in TIC rats (Fig. 7a–e and Additional file 1: Fig. S3a–h). In addition, Trz caused cardiomyocyte pyroptosis as indicated by increased expression of NLRP3 and cleaved Gasdermin D/total Gasdermin D (Fig. 8a, b and Additional file 1: Fig. S4a–e). However, treatment with donepezil reduced cardiac pyroptosis as shown by alleviation of the expression of NLRP3 and cleaved Gasdermin D/total Gasdermin D in Trz-treated rats (Fig. 8a, b and Additional file 1: Fig. S4a–e). Additionally, Trz increased cardiomyocyte ferroptosis as indicated by increased ACLS4 expression when compared to the control group (Fig. 8c). Again, donepezil treatment attenuated ferroptosis by reducing ACLS4 expression in those TIC rats (Fig. 8c and Additional file 1: Fig. S4f, g). Interestingly, necroptosis was not upregulated in those Trz-treated rats (Fig. 8d–f and Additional file 1: Fig. S4h–s). Collectively, donepezil alleviated cardiomyocyte death by reducing apoptosis, pyroptosis, and ferroptosis in Trz-treated rats.

### Donepezil protected against Trz-induced cytotoxicity in vitro via mAChR activation

**Fig. 9:**
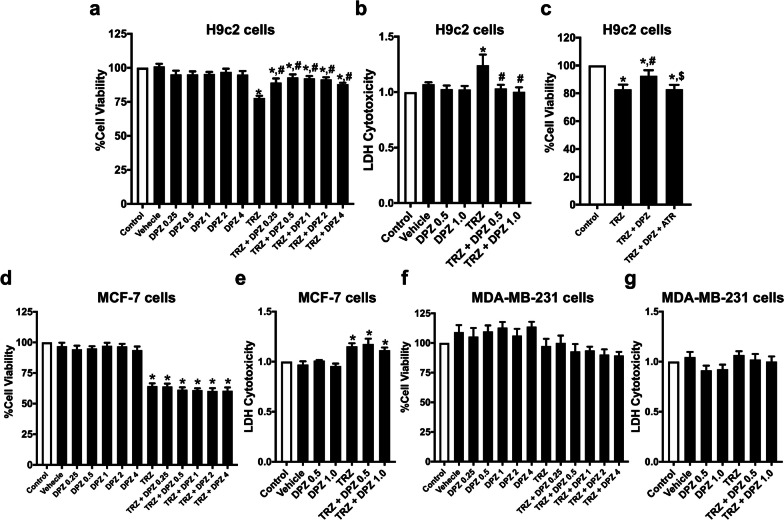
The effects of donepezil on cytotoxicity in cardiomyoblast and breast cancer cells treated with Trz. **a** Cell viability and **b** cytotoxicity of H9c2 cells; **c** Atropine, a muscarinic acetylcholine receptor antagonist was applied in H9c2 cells treated with Trz with or without donepezil 0.5 μM for 48 h; **d** Cell viability and **e** cytotoxicity of MCF-7 cells; **f** Cell viability and **g** cytotoxicity of MDA-MB-231 cells. *ATR* atropine; *DPZ* donepezil; *Trz* trastuzumab. *n* = 5 per group. Data are presented as the mean ± SEM; *p < 0.05 vs. control; ^#^p < 0.05 vs. Trz; ^$^p < 0.05 vs. Trz + DPZ

To evaluate the underlying mechanism of donepezil, we measured the cell viability and cytotoxicity of Trz-treated cardiomyoblast H9c2 cells with or without donepezil treatment (Fig. 9a). As expected, Trz markedly reduced cell viability and increased LDH release in H9c2 cells (Fig. 9a, b). Co-treatment with donepezil attenuated the reduction of cell viability and reduced LDH levels in Trz-treated cells (Fig. 9a, b). However, atropine, a muscarinic AChR antagonist abrogated the cytoprotective effect of donepezil (Fig. 9c). Finally, the impact of donepezil on the anti-cancer efficacy of Trz was determined in both breast cancer cell lines (Fig. 9d–g). The results demonstrated that Trz decreased MCF-7 cell viability and increased LDH cytotoxicity (Fig. 9d, e). However, Trz did not induce MDA-MB-231 cell death and cytotoxicity (Fig. 9f, g). Donepezil treatment at all doses did not change the anti-cancer effect of Trz (Fig. 9d, e). Taken together, donepezil protected H9c2 cells via mAChR activation without lowering the anti-cancer efficacy of Trz in breast cancer cells.

## Discussion

In this study, we demonstrated that Trz-treated hearts showed an upregulation of oxidative stress and inflammation. Trz also induced mitochondrial dysfunction, mitochondrial dynamic imbalance, and impaired autophagy, resulting in cardiomyocyte death and LV dysfunction. Interestingly, we showed that donepezil attenuated cardiac injury and cardiac dysfunction induced by Trz, which is associated with (i) antioxidant, (ii) anti-inflammation, (iii) anti-apoptosis, (iv) anti-pyroptosis, (v) anti-ferroptosis, (vi) attenuation of autophagy impairment, and (vii) the protection of the cardiac mitochondria. At the mechanistic level, we identified that the cardioprotective effects of donepezil were linked to the activation of mAChR. Furthermore, donepezil did not reduce the anti-cancer effects of Trz in breast cancer cell lines.

Trz has been commonly used in combination with other chemotherapeutic agents, such as doxorubicin, to reduce mortality in various malignancies (Alsina et al. 2022; Vega Cano et al. 2022; Bando et al. 2023; Piccart-Gebhart et al. 2005; Mohan et al. 2018). Although doxorubicin and trastuzumab are anti-cancer drugs that could induce potentially life-threatening cardiotoxicity (Gorini et al. 2018), the mechanisms of cardiotoxic actions induced by these two anti-cancer drugs are different (Gorini et al. 2018; Shakir and Rasul 2009). The cardiotoxic phenotype of doxorubicin is dose-dependent irreversible cardiac dysfunction, known as Type 1 cardiotoxicity (Gorini et al. 2018; Shakir and Rasul 2009; Rochette et al. 2015). The mechanisms of doxorubicin-induced cardiotoxicity are associated with numerous processes, including the interaction of doxorubicin with topoisomerase 2B, leading to double-stranded DNA breaks (Gorini et al. 2018; Shakir and Rasul 2009; Rochette et al. 2015). Doxorubicin directly produces reactive oxygen species (ROS) from its semiquinone intermediates and the Fenton reaction (Gorini et al. 2018; Shakir and Rasul 2009; Rochette et al. 2015). Overproduction of ROS induces mitochondrial dysfunction and cardiomyocyte death (Gorini et al. 2018; Shakir and Rasul 2009; Rochette et al. 2015). Unlike doxorubicin-induced cardiotoxicity (DIC), TIC demonstrated a distinct clinical phenotype, which is characterized by dose-independent reversible cardiac damage, known as Type 2 cardiotoxicity (Wu et al. 2022; Mohan et al. 2018). Currently, the precise mechanism of TIC remains unclear. Several studies suggested that the mechanisms of TIC are multifactorial, including HER2 signaling alteration, oxidative stress, inflammation, mitochondrial dysfunction, and impaired cellular metabolism (Wu et al. 2022; Kitani et al. 2019; Matsukawa et al. 2011; Sun et al. 2022; Mohan et al. 2018; Riad et al. 2008). Furthermore, Trz is not only used in combination with other chemotherapeutic agents, but is also used as a monotherapy, including in elderly patients with early HER2^+^ breast cancer (Sawaki et al. 2020; Konishi et al. 2022). Therefore, it is important to investigate the protective effects of donepezil not just in the DIC, but also in the TIC. These findings from our study will provide assurance that parasympathetic activation with donepezil could be a promising therapeutic approach not only for DIC patients, but also for TIC patients, and will also encourage further clinical investigations in those patients.

Noteworthily, TIC has been associated with cardiac sympathetic hyperactivity through modulating nitric oxide synthase and neuregulin/HER signaling in the rostral ventrolateral medulla (Lenneman et al. 2014; Guimaraes et al. 2015). In the present study, we demonstrated that Trz impaired HRV, indicating a sympathovagal imbalance. Importantly, Trz reduced parasympathetic activity by upregulating AChE activity. Therefore, enhancing parasympathetic activity by inhibiting AChE could be a promising therapeutic strategy for patients receiving chemotherapy. Several studies reported that inhibition of AChE exerted beneficial effects on autonomic function in various heart diseases including myocardial infarction and DIC (Khuanjing et al. 2021a; Fuente et al. 2013; Lataro et al. 2013). Here we demonstrated that donepezil effectively inhibited AChE activity, thus increasing parasympathetic activity in rats with TIC. In addition to enhanced parasympathomimetic activity, it has been shown that increased ACh levels upon AChE inhibition activated presynaptic mAChR of the sympathetic nerve, leading to decreased noradrenaline release (Manabe et al. 1991; Liu et al. 2019). At the cellular level, we demonstrated that these cardioprotective benefits were through the activation of cardiac mAChR, without altering the effects of Trz on cancer cells.

In the present study, the results showed that cardiac autonomic function was significantly impaired after Trz treatment, as indicated by the reduced HRV with a reduction in %LVEF. Although this change was not reflected in the maximum time derivative of LV pressure (dP/dt_max_), %LVEF was significantly reduced in Trz-treated rats, suggesting the reduction of contractility. These controversial findings could be due to the nature of the methods used for the assessment of LV systolic function. A previous study demonstrated that %LVEF is more sensitive and reliable in detecting systolic dysfunction than dP/d*t*_max_ in myocardial infarction pigs (Ishikawa et al. 2012). In that study, they found that both %LVEF and dP/d*t*_max_ showed a comparable relative decrease in severe LV dysfunction in pigs (Ishikawa et al. 2012). However, the relative decline of dP/d*t*_max_ is smaller than that of %LVEF when cardiac function is less impaired (Ishikawa et al. 2012). In our study, we found that Trz-treated rats showed a small but significant decrease in %LVEF (~ 11% reduction), compared with the control group, suggesting a small decline in contractility. Therefore, a decrease in dP/d*t*_max_ may not be observed in our study. According to the results of a cardiac injury biomarker, cardiac troponin I (cTnI) was slightly increased in the Trz-treated rats. An increase in cTnI levels was found to be associated with cardiomyocyte death (Hammarsten et al. 2018). In this study, we found an increase in cell death after Trz treatment in rat’s hearts. In addition, the MTT result from the in vitro study showed that the amount of H9c2 cell death was small (approximately 22.14%) in the Trz-treated group. In the case of LV function, Trz-treated rats showed a small but significant decrease in %LVEF (~ 11% reduction). For this reason, a slight rise in cTnI levels has been linked to a minor amount of cardiomyocyte death, leading to a mild impairment in cardiac function in rats after treatment with Trz.

It has been reported that cardiac inflammation plays a critical role in the development of the TIC (Kabel and Elkhoely 2017; Riad et al. 2008). Our study confirmed that Trz increased pro-inflammatory cytokine production as indicated by increased IL-6 and TNF-α mRNA expression. Moreover, we showed that donepezil suppressed cardiac inflammation in rats with TIC. It is well known that the cholinergic anti-inflammatory pathway plays a significant role in regulating inflammation in heart diseases (Khuanjing et al. 2020a; Lu and Wu 2021; Yuan et al. 2018). Mechanistically, activation of α7nAchR stimulates the Janus kinase 2/signal transducers and activators of transcription 3/NF-κB, leading to decreased pro-inflammatory cytokine production from cytokine-producing cells (Lu and Wu 2021). In line with our report, a previous study showed that donepezil reduced inflammation and improved cardiac function via activation of α7nAchR in the heart transplantation-induced I/R injury (Yuan et al. 2018).

Emerging evidence suggested that programmed cell death pathways play an essential role in the development of chemotherapy-related cardiotoxicity, in particular TIC (Ma et al. 2020; Sun et al. 2022; Kabel and Elkhoely 2017; Riccio et al. 2018; Re et al. 2019). Previous studies reported that Trz increased cardiomyocyte death through upregulation of various programmed cell death pathways, including apoptosis and ferroptosis (Sun et al. 2022; Kabel and Elkhoely 2017). However, other programmed cell death pathways (i.e., pyroptosis and necroptosis) have not been investigated. It is well known that the underlying mechanisms of TIC are mainly described via inhibition of HER2 signaling in the cardiomyocytes (Mohan et al. 2018). Generally, HER2 signaling plays a significant role in survival and apoptosis inhibition through stimulation of the phosphoinositide 3-kinase (PI3K)/protein kinase B (Akt) signaling pathway (Varga et al. 2015). Once activated, PI3K/Akt increased the expression of anti-apoptotic proteins (i.e., Bcl-2 and Bcl-xL) and downregulated pro-apoptotic proteins, B-cell lymphoma-extra small (Bcl-xS) and Bax (Wu et al. 2022; Mohan et al. 2018). On the other hand, inhibition of HER2 signaling by Trz diminished Bcl-xL and upregulated Bcl-xS, which promoted the intrinsic mitochondrial apoptosis pathway (Gorini et al. 2018; Mohan et al. 2018). Consistently, our data demonstrated that Trz increased the apoptotic markers, including the ratio of Bax/Bc-2, cytochrome c, and the ratio of cleaved caspase 3/caspase 3. Notably, donepezil cotreatment effectively attenuated apoptosis in rats with TIC. Previous studies reported that donepezil suppressed apoptosis in various heart diseases, by stimulating the pro-survival pathways (Handa et al. 2009; Khuanjing et al. 2020b).

In addition to apoptosis, cardiac pyroptosis markers were also upregulated in rats with TIC. It has been known that pyroptosis activation is mediated by the NLRP3/caspase 1 pathway (Re et al. 2019). In this study, we found that Trz upregulated cardiomyocyte pyroptosis by increasing NLRP3 and cleaved Gasdermin D/Gasdermin D, which was abrogated by donepezil treatment. A possible mechanism of the anti-pyroptotic effect of donepezil could be through α7nAchR activation, which inhibits the NLRP3 inflammasome (Jiang et al. 2019). In addition, the anti-pyroptotic effect of donepezil may involve its anti-oxidative effect since NLRP3 could be activated by ROS (Zhou et al. 2011). In this study, we showed that donepezil alleviated ROS production as indicated by decreased mitochondrial ROS production and MDA levels. Previous studies showed that mAChR activation mitigated ROS levels in the hypoxia/reoxygenation of H9c2 cells and the ischemic hearts (Kong et al. 2012; Miao et al. 2013; Sun et al. 2014). In addition, AMPK activation reduced ROS levels by inhibiting the protein kinase C/NADPH oxidase pathway (Kong et al. 2012; Ceolotto et al. 2007). In connection, these findings may also explain why donepezil alleviates cardiac ferroptosis upon Trz treatment (Kong et al. 2012; Ceolotto et al. 2007).

Cardiac ferroptosis is also upregulated in Trz-treated hearts, as evidenced by increased lipid peroxidation and ACSL4 expression, which were counteracted by donepezil treatment. Mechanistically, increased lipid peroxidation in Trz-treated hearts may be attenuated by the antioxidative effects of donepezil as described above. Furthermore, a previous study reported that α7nAchR activation suppressed ferroptosis in mice with acute respiratory distress syndrome and LPS-treated alveolar epithelial cells (Zhang et al. 2022). However, the underlying mechanism of the anti-ferroptosis effects of donepezil in the heart is largely unknown and remains to be elucidated. It is interesting to note that necroptosis was not upregulated by Trz treatment. We speculated that necroptosis may take a longer duration to respond to Trz treatment. A previous study reported that necroptosis was upregulated a week after myocardial infarction (Zhang et al. 2020). In this study, we euthanized the rats to collect heart tissue 5 days after the last dose of Trz injection. Therefore, necroptosis may not be upregulated at that time. However, the association between TIC and necroptosis is still largely unknown and will need further investigation in the future.

Autophagy is a crucial process for cellular homeostasis under normal and stressful conditions (Mei et al. 2015). The autophagic process is mainly regulated by the AMPK/mTOR/ULK-1 signaling (Mei et al. 2015). Once activated, AMPK increased autophagy by stimulating ULK-1 and inhibiting mTOR-mediated autophagy (Mei et al. 2015). A previous study demonstrated that Trz increased mitochondrial dysfunction and suppressed the autophagic process (Kitani et al. 2019). In that study, Trz reduced AMPK activation and activated mTOR signaling, subsequently suppressing autophagic activity (Kitani et al. 2019). Consistently, our study showed that Trz decreased AMPK phosphorylation and autophagy-related proteins. Donepezil effectively attenuated the reduction of phosphorylated AMPK, resulting in the reactivation of AMPK and autophagy. In the cardiac I/R injury model, donepezil was shown to attenuate cardiac injury by improving autophagy (Khuanjing et al. 2021b). Furthermore, it has been shown that ACh activated the mAChR/AMPK/mTOR pathway, thus restoring autophagy and protecting H9c2 cells in the H/R injury model (Zhao et al. 2013). Collectively, this implied that donepezil possibly preserved autophagy by activation of the mAChR/AMPK/mTOR pathway.

It is well established that mitochondrial dysfunction is related to cardiomyocyte death and cardiac dysfunction in TIC (Gorini et al. 2018; Varga et al. 2015; Kitani et al. 2019; Sun et al. 2022). In this study, we showed that Trz increased mitochondrial ROS levels, MMP changes, and mitochondrial swelling. Increased ROS levels upon Trz treatment also upregulated Drp-1 activation and increased translocation to the mitochondria, subsequently inducing the mitochondrial fission process. In addition, we demonstrated that Trz reduced mitochondrial fusion protein, leading to mitochondrial dynamic imbalance. On the other hand, our results showed that donepezil treatment attenuated cardiac mitochondrial dysfunction and rebalanced mitochondrial dynamics in Trz-treated rats. It has been reported that an AChE inhibitor reduces mitochondrial dysfunction and mitochondrial dynamic imbalance in several heart diseases including cardiac I/R injury, DIC, and high-fat diet-induced cardiomyopathy (Khuanjing et al. 2020b, 2021a; Lu et al. 2018). The possible mechanism to protect the mitochondria is mostly related to the mAChR activation (Kong et al. 2012; Miao et al. 2013; Sun et al. 2014). Once activated, it activates forkhead box subfamily O3a/peroxisome proliferator-activated receptor γ co-activator 1α, resulting in alleviated mitochondrial dysfunction and ROS levels (Sun et al. 2014). In the ischemic hearts, it has been shown that electrical vagus nerve stimulation activated mAChR/Ca^2+^/calmodulin-dependent protein kinase kinase-beta/AMPK signaling, leading to an attenuation of the mitochondrial dynamic imbalance (Xue et al. 2017).

It should be noted that the effectiveness of Trz in killing breast cancer cell lines was not compromised by the co-incubation of donepezil. This strengthened the effectiveness and safety of donepezil for the potential treatment of TIC in cancer patients. However, the role of donepezil on the anti-cancer efficacy of Trz should be further investigated in in vivo and in a clinical study. In addition, in this study, we did not use primary cardiomyocytes to evaluate the cytotoxic effects of Trz and the cytoprotective effects of donepezil. Primary cardiomyocytes either adult or neonatal rat ventricular cardiomyocytes are commonly used in many in vitro studies due to their physiological functions, including contractility and electrical properties (Abi-Gerges et al. 2020; Leone and Engel 2021). Although primary cardiomyocytes showed many benefits in cardiovascular research, their unstable isolation efficiency and extremely fragile nature still limited their use (Zhou et al. 2022; Kaur and Dufour 2012). On the other hand, H9c2 cells are alternatively useful for in vitro experiments and are commonly used in several studies (Sun et al. 2022; Watkins et al. 2011; Ding et al. 2023; Lee et al. 2022; Pecoraro et al. 2020; Belmonte et al. 2015). H9c2 cells demonstrated several similarities to primary cardiomyocytes, including membrane morphology, electrophysiological properties, and protein/metabolic signatures (Branco et al. 2015; Hescheler et al. 1991; Sipido and Marban 1991). Furthermore, cell line usage provides several advantages, such as being a cost-effective, consistent sample, and producing reproducible results (Kaur and Dufour 2012). Therefore, H9c2 cells were used to investigate various cardiotoxicity models, most notably the TIC (Sun et al. 2022; Watkins et al. 2011; Ding et al. 2023; Lee et al. 2022; Pecoraro et al. 2020; Belmonte et al. 2015). For these reasons, H9c2 cells were used to study cytotoxicity in our study. In addition, previous studies showed that the administration of Trz increased the infiltration of the cardiac tissues with inflammatory cells (Kabel and Elkhoely 2017; Yousif and Al-amran 2011). Although we found that Trz markedly increased the mRNA and protein expression of proinflammatory cytokines, including IL-6 and TNF-⍺, which were blunted by donepezil treatment, it is still important to investigate the effect of donepezil on inflammatory cell infiltration in Trz-treated hearts since donepezil effectively reduced immune cell infiltration in the heart transplantation-induced I/R injury (Yuan et al. 2018). However, we were unable to conduct immunohistochemistry/immuno fluorescent staining to evaluate immune cell infiltration in the heart, due to the limited cardiac tissue samples. Lastly, in the current study, we used only male rats, which might not recapitulate the clinical setting since Trz-treated patients consist of both males and females (Alsina et al. 2022; Vega Cano et al. 2022; Bando et al. 2023). Future studies should attempt to address whether a difference in the gender of rats influences the cardioprotective effects of donepezil.

## Conclusions

Donepezil protected hearts against TIC by reducing mitochondrial dysfunction, inflammation, autophagy disorder, and cardiomyocyte death, resulting in decreased LV dysfunction without lowering the anti-cancer efficacy of Trz. These promising findings suggest that inhibition of AChE by donepezil may be investigated as a novel therapeutic intervention in patients with TIC.

### Supplementary Information

**Additional file 1: Table S1.** List of antibodies used in this study. **Table S2.** List of primers used in this study. **Figure S1.** Western blot images of OPA1, Mfn1, p-drp1^ser616^, Drp1, VDAC, and GAPDH for Fig. 5. **Figure S2.** Western blot images of Beclin-1, LC3-II/I, p62, PINK1, Parkin, p-AMPKα^Thr172^, AMPKα, and β-actin for Fig. 6. **Figure S3.** Western blot images of Cleaved Caspase 3, Caspase 3, Cytochrome *c*, Bax, Bcl-2, and β-actin for Fig. 7. **Figure S4.** Western blot images of NLRP3, Cleaved Gasdermin D, ACSL4, RIP1, p-RIP3^ser232^, RIP3, p-MLKL^ser358^, MLKL, and β-actin for Fig. 8.

## Data Availability

All data and materials are provided in the manuscript. Additional data and materials are available upon reasonable request.

## Abbreviations

ACh: Acetylcholine
AChE: Acetylcholinesterase
AMPKα: AMP-activated protein kinase alpha
Bax: Bcl-2-associated X
Bcl-2: B-cell lymphoma 2
cDNA: Complementary deoxyribonucleic acid
cTnI: Cardiac troponin I
dP/dt_max_: Maximum time derivative of LV pressure
dP/dt_min_: Minimum time derivative of LV pressure
Drp1: Dynamin-related protein 1
E/A: Early filling to atrial filling velocities
ELISA: Enzyme-linked immunosorbent assay
GAPDH: Glyceraldehyde 3-phosphate dehydrogenase
HF: High-frequency component
HPLC: High-performance liquid chromatography
HRV: Heart rate variability
I.P.: Intraperitoneal injection
IL-6: Interleukin 6
LC3B: Light chain 3B
LDH: Lactate dehydrogenase
LF: Low frequency component
LV: Left ventricular
LVEDP: LV end-diastolic pressure
LVEF: LV ejection fraction
LVESP: LV end-systolic pressure
LVFS: LV fractional shortening
mAChR: Muscarinic ACh receptor
MDA: Malondialdehyde
Mfn1: Mitofusin 1
MLKL: Mixed-lineage kinase domain-like
MTT: 3-(4,5-Dimethylthiazol-2-yl)-2,5-diphenyltetrazolium bromide
NT-proBNP: N-terminal pro b-type natriuretic peptide
OPA1: Optic atrophy 1
P–V loop: Pressure–volume loop
p62 (SQSTM1): Sequestosome 1
PINK1: PTEN-induced putative kinase 1
RIP1: Receptor-interacting protein kinase 1
RIP3: Receptor-interacting protein kinase 3
ROS: Reactive oxygen species
SV: Stroke volume
TBARS: Thiobarbituric acid reactive substances
TNF-α: Tumor necrosis factor-α
TNFR1: TNF receptor 1
Trz: Trastuzumab
TIC: Trastuzumab-induced cardiotoxicity
TUNEL: Terminal deoxynucleotidyl transferase dUTP nick end labeling
VDAC: Voltage-dependent anion channel
