# Supplementary material for: Acetylcholinesterase inhibition protects against trastuzumab-induced cardiotoxicity through reducing multiple programmed cell death pathways
Source: Mol Med. 2023 Sep 11;29:123. doi: 10.1186/s10020-023-00686-7 (PMC10494358; doi:10.1186/s10020-023-00686-7)
Supplement: Supplementary file 1 — Additional file 1: Table S1. List of antibodies used in this study. Table S2. List of primers used in this study. Figure S1. Western blot images of OPA1, Mfn1, p-drp1ser616, Drp1, VDAC, and GAPDH for Fig. 5. Figure S2. Western blot images of Beclin-1, LC3-II/I, p62, PINK1, Parkin, p-AMPKαThr172, AMPKα, and β-actin for Fig. 6. Figure S3. Western blot images of Cleaved Caspase 3, Caspase 3, Cytochrome c, Bax, Bcl-2, and β-actin for Fig. 7. Figure S4. Western blot images of NLRP3, Cleaved Gasdermin D, ACSL4, RIP1, p-RIP3ser232, RIP3, p-MLKLser358, MLKL, and β-actin for Fig. 8. [file 10020_2023_686_MOESM1_ESM.docx]

**Additional Tables**

**Additional Table S1. List of antibodies used in this study**

| **No** | **Antibody** | **Providers** | **Catalog Number** | **Dilution** | **Host** |
| --- | --- | --- | --- | --- | --- |
| 1 | Anti-ACLS4 | Santa cruz biotechnology, INC. | sc-365230 | 1:1,000 | Mouse |
| 2 | Anti-AMPKα | Cell signaling | 2532 | 1:1,000 | Rabbit |
| 3 | Anti-Bax | Cell signaling | 2772 | 1:1,000 | Rabbit |
| 4 | Anti-Bcl-2 | Abcam | ab196495 | 1:1,000 | Rabbit |
| 5 | Anti-Beclin-1 | Cell signaling | 3495 | 1:1,000 | Rabbit |
| 6 | Anti-Cleaved Gasdermin D | Cell signaling | 10137 | 1:1,000 | Rabbit |
| 7 | Anti-Caspase-3 | Cell signaling | 14220 | 1:1,000 | Rabbit |
| 8 | Anti-Cytochrome *c* | Cell signaling | 4272 | 1:1,000 | Rabbit |
| 9 | Anti-Drp1 | Cell signaling | 5391 | 1:1,000 | Rabbit |
| 10 | Anti-GAPDH | Abcam | ab181602 | 1:1,000 | Rabbit |
| 11 | Anti-Gasdermin D | Cell signaling | 39754 | 1:1,000 | Rabbit |
| 12 | Anti-IL-6 | Abcam | Ab9324 | 1:1,000 | Mouse |
| 13 | Anti-LC3A/B | Cell signaling | 12741 | 1:1,000 | Rabbit |
| 14 | Anti-Mitofusin-1 | Abcam | ab104274 | 1:1,000 | Rabbit |
| 15 | Anti-MLKL | Invitrogen | PA-43960 | 1:1,000 | Rabbit |
| 16 | Anti-OPA1 | Cell signaling | 80471 | 1:1,000 | Rabbit |
| 17 | Anti-p62/SQSTM1 | Cell signaling | 5114 | 1:1,000 | Rabbit |
| 18 | Anti-Parkin | Abcam | Ab77924 | 1:1,000 | Rabbit |
| 19 | Anti-Phospho-AMPKα (Thr172) | EMD millipore | 07-681 | 1:1,000 | Rabbit |
| 20 | Anti-Phospho-Drp1 (Ser616) | Cell signaling | 3455 | 1:1,000 | Rabbit |
| 21 | Anti-Phospho-MLKL (Ser358) | Invitrogen | PA5-105678 | 1:1,000 | Rabbit |
| 22 | Anti-Phospho-RIP1 (Ser166) | Cell signaling | 31122 | 1:1,000 | Rabbit |
| 23 | Anti-Phospho-RIP3 (Ser232) | Abcam | ab195117 | 1:1,000 | Rabbit |
| 24 | Anti-PINK-1 | Abcam | ab23707 | 1:1,000 | Rabbit |
| 25 | Anti-RIP1 | Cell signaling | 3493 | 1:1,000 | Rabbit |
| 26 | Anti-RIP3 | Cell signaling | 15828 | 1:1,000 | Rabbit |
| 27 | Anti-Total DRP1 | Cell signaling | 5391 | 1:1,000 | Rabbit |
| 28 | Anti-TNF-⍺ | Abcam | Ab205587 | 1:1,000 | Rabbit |
| 29 | Anti-VDAC | Cell signaling | 4661 | 1:1,000 | Rabbit |
| 30 | Anti-β-Actin | Santa cruz biotechnology, INC. | sc-47778 | 1:1,000 | Mouse |
| 31 | Anti-mouse IgG, HRP-linked antibody | Cell signaling | 7076 | 1:1,000 | Horse |
| 32 | Anti-rabbit IgG, HRP-linked antibody | Cell signaling | 7074 | 1:1,000 | Goat |

**Additional Table S2. List of primers used in this study**

| **Gene** | **Forward primer (5′–3′)** | **Reverse primer (5′–3′)** |
| --- | --- | --- |
| *Tnf-a* | ACTCCCAGAAAAGCAAGCAA | CGAGCAGGAATGAGAAGAGG |
| *Il-6* | TCCTACCCCAACTTCCAATGCTC | TTGGATGGTCTTGGTCCTTAGCC |
| *β-actin* | GGAGATTACTGCCCTGGCTCCTA | GACTCATCGTACTCCTGCTTGCTG |

**Additional Figures**

**Additional Fig. S1**

**
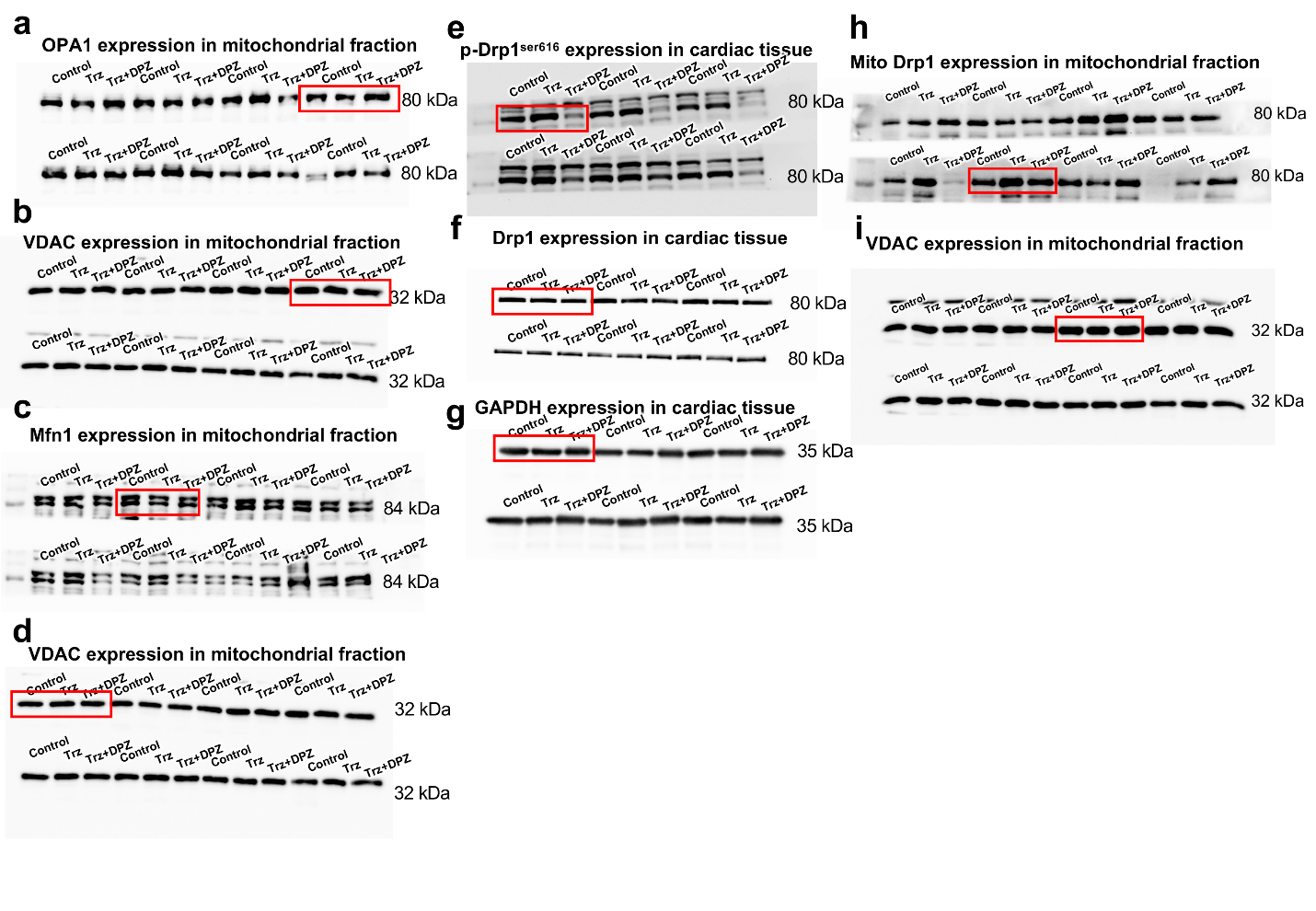
 Additional Fig. S1. Western blot images of OPA1, Mfn1, p-drp1^ser616^, Drp1, VDAC, and GAPDH for Fig. 5.** (a) OPA1 and (b) VDAC in mitochondrial fraction for Fig. 5e, (c) Mfn1 and (d) VDAC in mitochondrial fraction for Fig. 5f, (e) p-drp1^ser616^, (f) Drp1, and (g) GAPDH in cardiac tissue for Fig. 5g, (h) mitochondrial Drp1 and (i) VDAC in mitochondrial fraction for Fig. 5h. The red boxes present the representative band selected for Fig. 5.

**Additional Fig. S2**

**
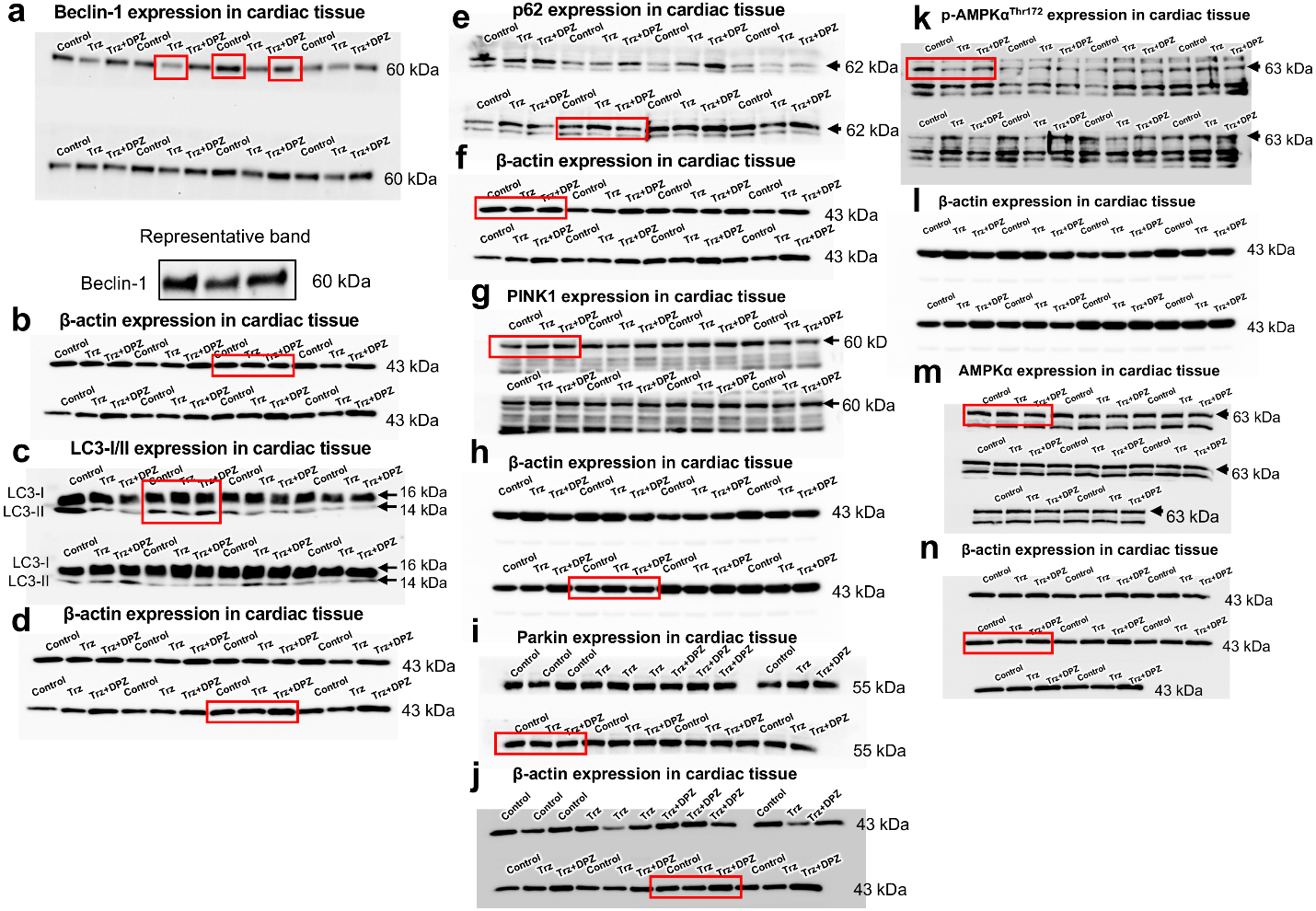
**

**Additional Fig. S2. Western blot images of Beclin-1, LC3-II/I, p62, PINK1, Parkin, p-AMPKα^Thr172^, AMPKα, and β-actin for Fig. 6.** (a) Beclin-1 and (b) β-actin in cardiac tissue for Fig. 6a, (c) LC3-II/I and (d) β-actin in cardiac tissue for Fig. 4b, (e) p62 and (f) β-actin in cardiac tissue for Fig. 6d, (g) PINK1 and (h) β-actin in cardiac tissue for Fig. 6e, (i) Parkin and (j) β-actin in cardiac tissue for Fig. 6f, (k) p-AMPKα^Thr172^, (l) β-actin, (m) AMPKα, and (n) β-actin in cardiac tissue for Fig. 6g. The red boxes present the representative band selected for Fig. 6.

**Additional Fig. S3**

**
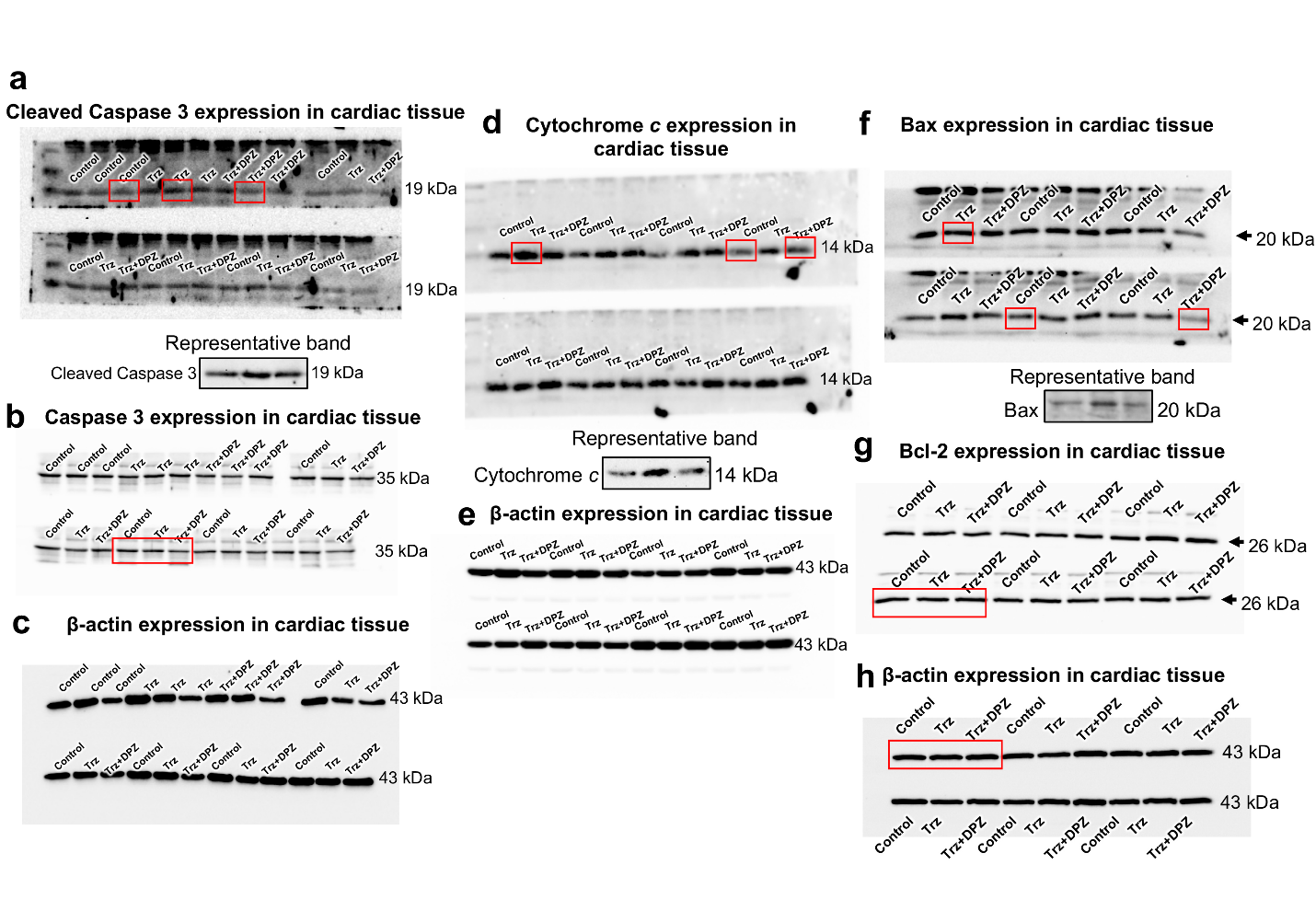
**

**Additional Fig. S3. Western blot images of Cleaved Caspase 3, Caspase 3, Cytochrome *c*, Bax, Bcl-2, and β-actin for Fig. 7.** (a) Cleaved Caspase 3, (b) Caspase 3, (c) β-actin, (d) Cytochrome *c*, (d) β-actin, (f) Bax, (g) Bcl-2, and (h) β-actin in cardiac tissue for Fig. 7a. The red boxes present the representative band selected for Fig. 7.

**Additional Fig. S4**

**
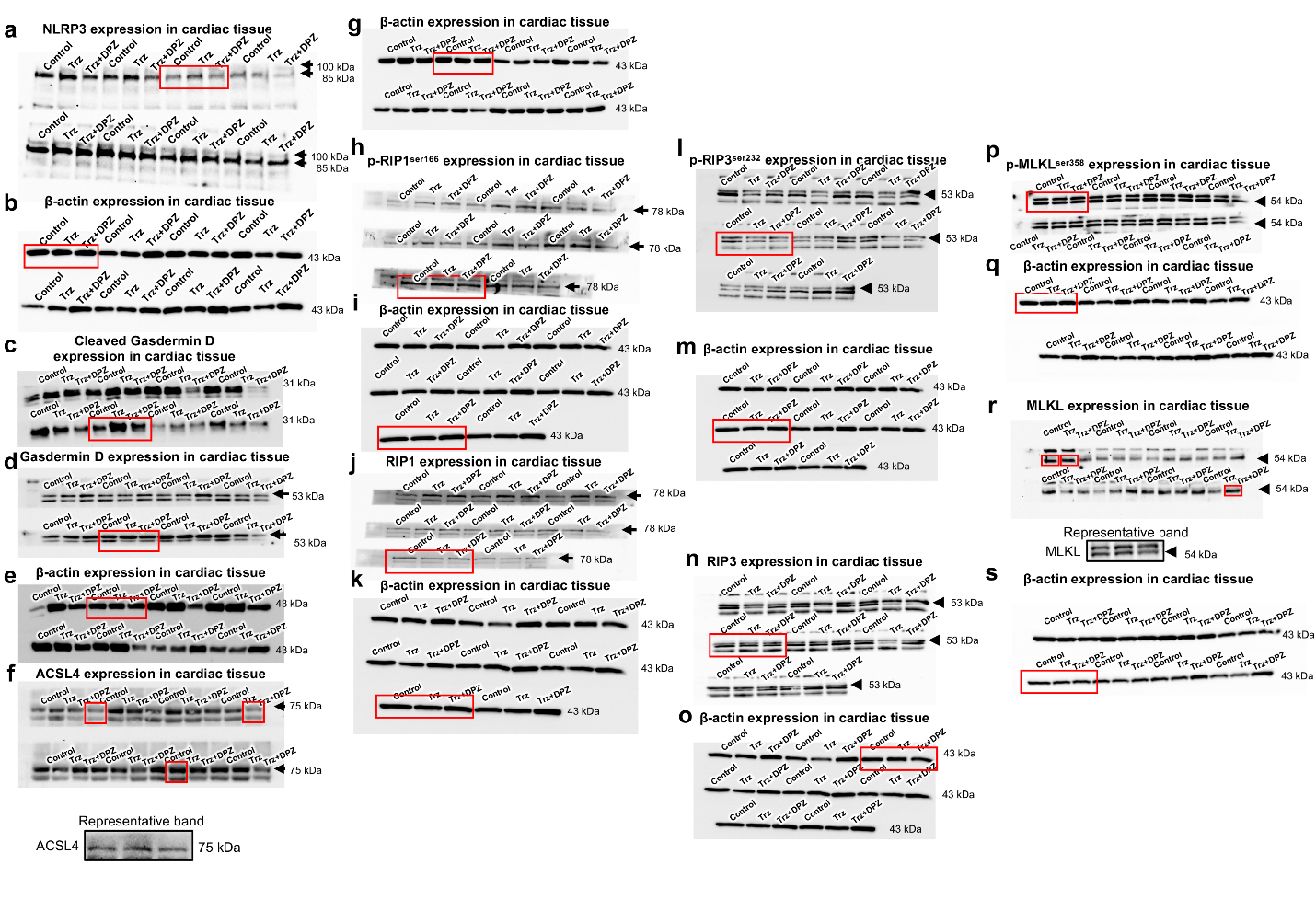
**

**Additional Fig. S4. Western blot images of NLRP3, Cleaved Gasdermin D, ACSL4, RIP1, p-RIP3^ser232^, RIP3, p-MLKLser358, MLKL, and β-actin for Fig. 8.** (a) NLRP3 and (b) β-actin in cardiac tissue for Fig. 8a, (c) Cleaved Gasdermin D, (d) Gasdermin D, (e) β-actin in cardiac tissue for Fig. 8b, (f) ACSL4 and (g) β-actin in cardiac tissue for Fig. 8c, (h) p-RIP1^ser166^, (i) β-actin, (j) RIP1, and (k) β-actin in cardiac tissue for Fig. 8d, (l) p-RIP3^ser232^, (m) β-actin, (n) RIP3, and (o) β-actin in cardiac tissue for Fig. 8e, (p) p-MLKL^ser358^, (q) β-actin, (r) MLKL, and (s) β-actin in cardiac tissue for Fig. 8f. The red boxes present the representative band selected for Fig. 8.
